# Fully Polarimetric Radar Cross‐Section and High‐Resolution Range Profile Co‐Modulation via Diversely Time‐Modulated Metasurface

**DOI:** 10.1002/nap2.70205

**Published:** 2026-07-14

**Authors:** Hantao Xu, Jinxing Guo, Xinyang Mao, Shijian Yu, Yihao Xu, Peng You, Bin Zheng, Dongfang Guan, Zhen Liu, Yongxiang Liu

**Affiliations:** ^1^ College of Electronic Science and Technology National University of Defense Technology Changsha China; ^2^ Hangzhou Global Scientific and Technological Innovation Center Zhejiang University Hangzhou China

**Keywords:** broadband, full polarizations, high‐resolution range profile (HRRP), radar cross section (RCS), radar jamming, time‐modulated metasurfaces

## Abstract

Advanced polarimetric radar systems, capable of capturing the full scattering matrix of targets, present significant challenges to electronic countermeasures (ECM). Although programmable metasurfaces offer a platform for electromagnetic manipulation, existing designs struggle to achieve simultaneous, diverse control over fully polarimetric radar cross section (RCS) and high‐resolution range profile (HRRP). To address these limitations, a novel diversely time‐modulated metasurface (DTMM) is proposed. The integrated two anti‐parallel PIN diodes in each DTMM element enable reconfigurable polarization conversion and wideband stable 1‐bit phase shift. A specially engineered time‐varying circuit enables the generation of unipolar/bipolar pulsed voltages with customizable amplitudes, facilitating diversely temporal modulation. Based on this architecture, we further establish the corresponding time‐modulated theory and demonstrate its fully polarimetric RCS and HRRP co‐modulation capability. Experimental results from a fabricated prototype show well agreement with simulations and theory, validating its ability to generate diverse jamming effects. This work provides a reliable and versatile platform for countering new‐generation polarimetric radars, significantly advancing the radar jamming technology.

## Graphical abstract

A diversely time‐modulated metasurface enables broadband co‐modulation of fully polarimetric radar cross section and high‐resolution range profiles. By integrating anti‐parallel PIN diodes and programmable temporal coding, the platform dynamically redistributes scattering among HH/VV and HV/VH channels, generating tunable target weakening, false targets, and range‐domain interference for advanced polarimetric radar countermeasures.

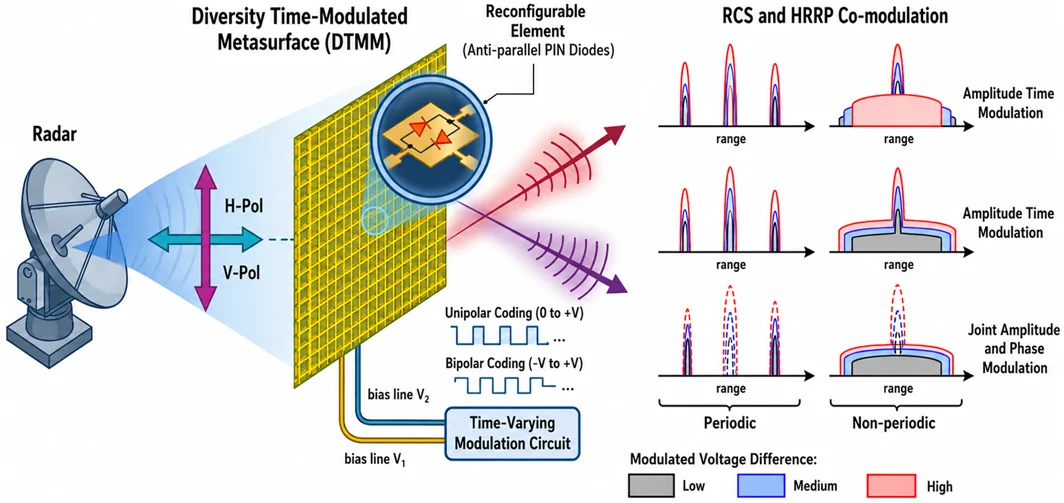

## 1 Introduction

With the advancement of semiconductor technology and radar signal processing techniques, modern polarimetric radars can acquire target information across multiple domains, including time, frequency, energy, and polarization [1, 2, 3, 4, 5]. This comprehensive information acquisition capability poses new challenges to electronic countermeasures (ECM), as jamming focused solely on a single polarization or signature is often performance‐limited and easily identified. Consequently, how to simultaneously control energy‐domain features like radar cross section (RCS), as well as frequency‐domain signatures such as high‐resolution range profile (HRRP) under each polarization state, has become a core challenge in advanced ECM.

Traditional radar ECM platforms mainly include two categories. The first one is passive jamming, such as corner reflectors, Luneburg lens reflector, chaff, and absorbing materials, which rely on fixed physical structures to attenuate or enhance echoes [6, 7, 8, 9, 10, 11]. Although reliable, this static approach lacks the adaptability required to counter agile detection systems in complex electromagnetic (EM) environments. Active jamming technologies based on digital radio‐frequency memory (DRFM) are another category. Although offering high‐fidelity coherent deception, this technique suffers from prohibitive costs, high power consumption, and complex hardware architecture [12, 13].

In recent years, programmable metasurfaces (PMs) have gained widespread attention in the field of radar ECM due to their advantages of lightweight and flexible modulation, while also avoiding the inherent conflicts between traditional active and passive jamming platforms [14, 15, 16, 17, 18, 19]. By integrating active lumped elements into subwavelength periodic arrays, PMs can dynamically control EM parameters in real time. By adjusting the matching state between surface impedance and free space, PMs can control the partial conversion of EM energy into thermal energy to control RCS [20]. Through spatial coding, PMs effectively manipulate the spatial energy distribution to control RCS [21, 22, 23]. However, these static methods can only control the real target's own RCS to achieve stealth or camouflage, and cannot produce false targets to deceive radar. Through temporal coding, PMs reshape the spectral response, enabling micro‐motion imitation, velocity deception, HRRP imitation, and even imaging jamming [24, 25, 26, 27, 28, 29]. Recent advances in time‐modulated metasurfaces (TMMs) have further expanded these capabilities. The underlying physical mechanism is that when a radar signal impinges on a metasurface with a time‐varying reflection coefficient, the reflected echo becomes temporally modulated. For a linear frequency modulation (LFM) radar waveform, this temporal modulation redistributes the echo energy after pulse compression, resulting in controllable changes in the HRRP. Periodic time modulation [30, 31] of amplitude produces discrete harmonic components, which appear as symmetrically distributed false targets in the range profile; pseudorandom time modulation [32] spreads the echo energy into continuous harmonic components, producing noise‐like interference strips around the real target. Phase time modulation [33, 34] can further transfer the energy of the central target peak to other harmonic components, enabling target self‐blanking. Moreover, sophisticated coding schemes like asynchronous and multi‐frequency modulation have significantly enhanced the degrees of freedom for signal processing, enabling finer control over HRRP features [35, 36, 37].

Despite these promising advancements, current metasurface‐based ECM systems face severe limitations hindering their deployment against advanced polarimetric radars. Structurally, traditional broadband phase‐modulated elements often introduce interdigital capacitors [38], magneto‐electric dipoles [39] and antenna‐circuit configurations [40], resulting in complex designs, and dispersion response affecting the stability of broadband phase modulation. Functionally, time‐domain modulation is generally limited to either amplitude or phase, operating in the periodic or aperiodic mode, with little exploration of diverse modulation [41]. In terms of polarization, most metasurfaces operate only under a single co‐polarized channel like horizontal‐horizontal (HH) or vertical‐vertical (VV). Even the few designs [42, 43] attempting dual‐channel control (HH and VV) often fail to address the complete manipulation of full polarization scattering matrix, including two cross‐polarized horizontal‐vertical (HV) and vertical‐horizontal (VH) channels. Furthermore, although RCS and HRRP represent the most critical radar signatures for target detection, their coordinated manipulation remains unexplored. Therefore, designing a diversely time‐modulated metasurface (DTMM) capable of full polarimetric RCS and HRRP co‐modulation presents a highly challenging problem.

To circumvent these limitations, we herein propose a novel broadband DTMM capable of simultaneous RCS and HRRP co‐modulation across full polarizations. Each DTMM unit integrates two anti‐parallel PIN diodes, enabling reconfigurable polarization conversion for both *x*‐ and *y*‐polarized waves while avoiding complex resonant design, as broadband stable phase shift is achieved simply by altering the direction of the induced current. A diversely polarimetric time‐varying theory is proposed to guide the coding design, supported by a customized time‐varying modulation circuit for the practical implementation. Figure 1 schematically illustrates the diverse radar jamming concept enabled by the proposed DTMM for full polarizations. A typical experimental scenario for radar jamming is constructed using the fabricated DTMM prototype. Temporal modulation of both amplitude and phase allows flexible switching to blank the peaks of the real target. Periodic and aperiodic coding sequences are employed to generate false targets and jamming strips in the HRRP, respectively. The experimental results show well agreement with simulations, demonstrating its significant application potential in ECM scenarios and advancing the development of radar jamming technology.

**FIGURE 1:**
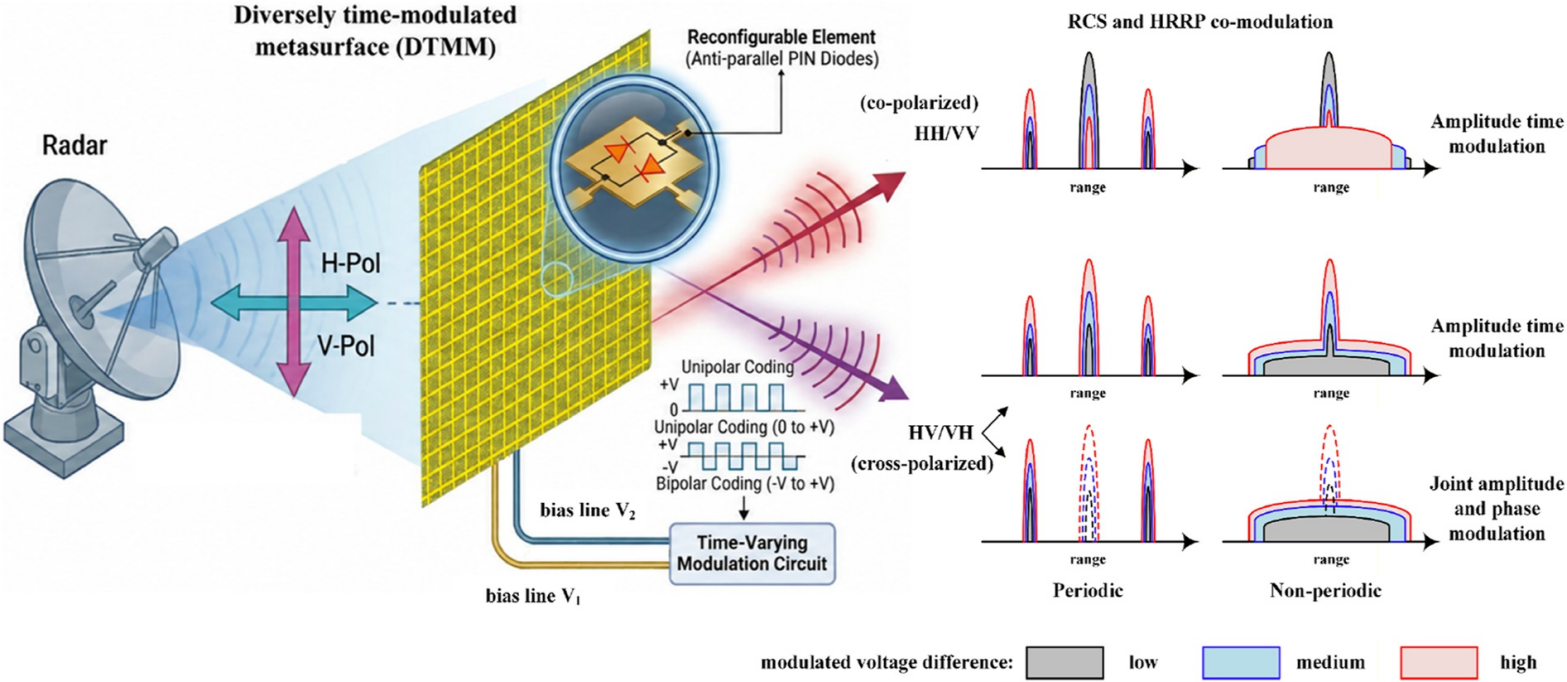
The schematic of the proposed DTMM for fully polarimetric jamming.

## 2 DTMM Design

### 2.1 DTMM Element Design

To design a DTMM for fully polarimetric jamming, it is crucial to develop a DTMM element capable of independently manipulating two orthogonally linear‐polarized EM waves while flexibly distributing energy between its co‐polarized and cross‐polarized conversion channels. Additionally, broadband co‐modulated jamming of RCS and HRRP requires simultaneous amplitude and phase control across a wide bandwidth. Conventional 45° axisymmetric polarization conversion elements inherently possess broadband and high‐efficiency orthogonal linear polarization conversion capability [44, 45]. Rotating such an element by 90° about its center, an ultra‐wideband and stable 180° reflection phase difference can be achieved in the cross‐polarized channel compared to its unrotated state [46].

Inspired by this, we propose a novel broadband DTMM element, which enables reconfigurable polarization conversion for dual linear polarizations and independent control of both amplitude and phase. The overall configuration of the proposed DTMM element is illustrated in Figure 2a. It features a sandwich structure comprising three metal layers and two dielectric layers. The top metal layer consists of four identical quasi‐dart‐shaped patches oriented toward the diagonals, serving to receive and radiate EM waves. The upper dielectric substrate is formed by laminating two layers of blue Rogers RO4350B with a red PP bonding film to achieve a specific thickness requirement. The bottom metal layer serves as the active control layer (see Figure 2b), integrating two anti‐parallel PIN diodes (Madp‐000907) designated as P_1_ and P_2_. These are modeled as a variable resistor *R*_*d*_ in parallel with the parasitic capacitance of 0.025 pF [47, 48]. Flexible functional switching is achieved by controlling the input voltage difference $U_{d}$ across terminals V_1_ and V_2_. Three lumped inductors (Coilcraft 0402DC‐4N3X) are used as RF chokes in the direct‐current (DC) bias network, thereby suppressing radio‐frequency (RF) leakage and reducing crosstalk between adjacent bias lines. The dimensional parameters of the DTMM element are: *p* = 9.48 mm, *r*_1_ = 1.90 mm, *r*_2_ = 3.57 mm, *w*_1_ = 2.60 mm, *w*_2_ = 0.70 mm, *l*_1_ = 0.28 mm, *l*_2_ = 0.55 mm.

**FIGURE 2:**
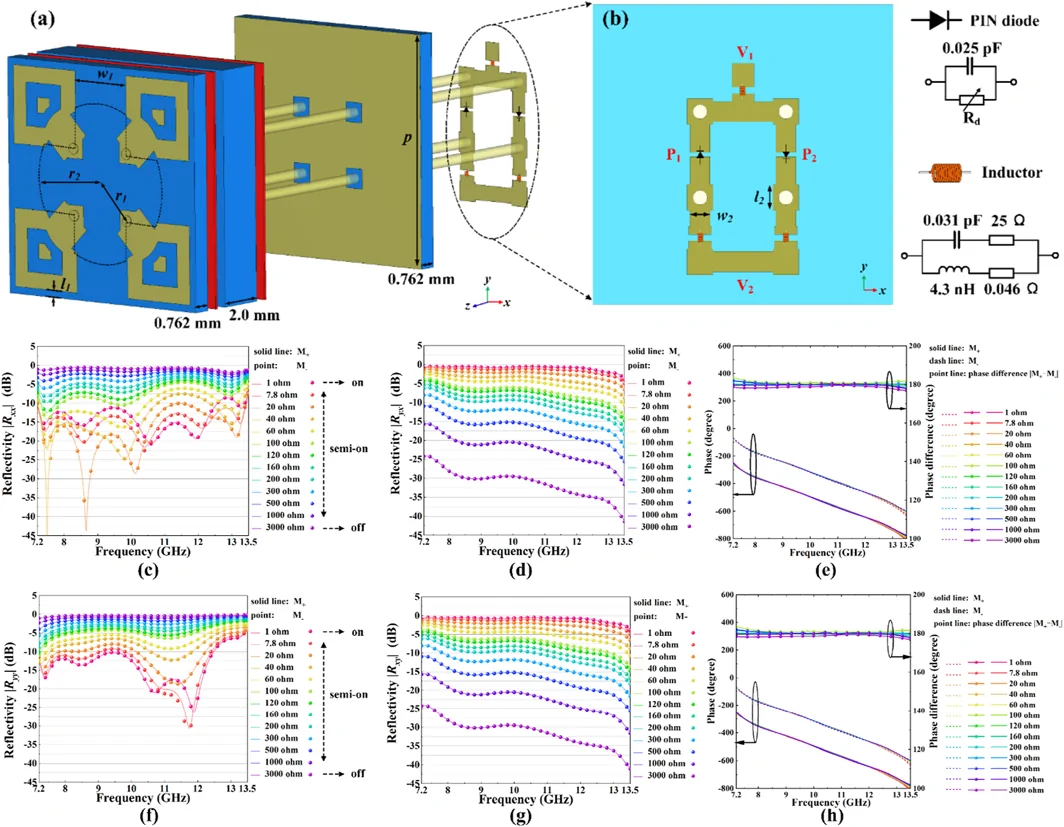
The structural design and simulated EM responses of the DTMM element. (a) Side view and (b) bottom view of the element. Amplitude tuning under *x*‐polarized incidence: (c) co‐polarized reflection |*R*_*xx*_| and (d) cross‐polarized reflection |*R*_*yx*_| as *R*_*d*_ varies. (e) Phase switching under *x*‐polarized incidence: cross‐polarized reflection phases *φ*_*yx*_ of *M*_+_ and *M*_−_, and their phase difference |Δ*φ*_*yx*_|. Amplitude tuning under *y*‐polarized incidence: (f) co‐polarized reflection |*R*_*yy*_| and (g) cross‐polarized reflection |*R*_*xy*_| as *R*_*d*_ varies. (h) Phase switching under *y*‐polarized incidence: cross‐polarized reflection phases *φ*_*xy*_ of *M*_+_ and *M*_−_, and their phase difference |Δ*φ*_*xy*_|. Here, *M*_+_ and *M*_−_ denote two opposite‐bias cross‐polarized conversion states, corresponding to the solid line and point in the amplitude tuning, and the solid line and dashed line in phase switching.

Simulated results in Figure 2c–h present the reflection responses of the DTMM element under both *x*‐ and *y*‐polarized incidence at varying resistance states. Due to the anti‐parallel configuration, one diode remains in the off‐state regardless of the input voltage variations. Specifically, when V_1_ equals V_2_, both diodes are non‐conducting, with the resistances of P_1_ and P_2_ approximately 3000 Ω. At this time, |*R*_*xx*_| and |*R*_*yy*_| exceed 0.9 from 7.2 to 13 GHz, whereas |*R*_*xy*_| and |*R*_*yx*_| remain below 0.1, indicating a fully co‐polarized reflection state *S*_*0*_. When $V_{1}<V_{2}$ and the unit is in state *M*_−_, as $U_{d}$ continues to increase, P_2_ remains in an OFF state, and the resistance of P_1_ decreases, progressively converting co‐polarized energy into the cross‐polarized domain until P_1_ becomes fully conducting. Consequently, |*R*_*xx*_| and |*R*_*yy*_| decrease, whereas |*R*_*xy*_| and |*R*_*yx*_| increase across the same frequency band. Similarly, when $V_{1}>V_{2}$ and the unit is in a state *M*_+_, as $U_{d}$ continues to increase, P_1_ remains in an OFF state, and the resistance of P_2_ decreases, progressively converting co‐polarized energy into the cross‐polarized domain until P_2_ becomes fully conducting. But, *M*_+_ maintains a stable 180° reflection phase difference in cross‐polarized domain from 7.2 to 13.5 GHz compared to *M*_−_, even if the resistance varies continuously (individual phase results are provided in Supporting Information S1). Even under oblique incidence, the reflection amplitude and phase responses of DTMM units in the polarization conversion state remain consistent, as detailed in Supporting Information S1. The aforementioned simulated results fully demonstrate the broadband amplitude and phase modulation capability of the DTMM element for full polarizations. The broadband cross‐polarized phase modulation mechanisms of DTMM units under *M*_+_ and *M*_−_ states are detailed in the simulated surface current analysis in Supporting Information S1. In addition, an explanation of the bandwidth and design shape of DTMM units is provided in Supporting Information S1.

It should be clarified that the full‐polarimetric modulation discussed in this work is defined with respect to a fixed pair of orthogonal linear polarization bases, namely the *x*/*y* basis of the metasurface or equivalently the H/V basis of the radar system. Under this basis, the proposed DTMM manipulates the four scattering‐matrix entries |*R*_*xx*_|, |*R*_*yy*_|, |*R*_*yx*_|, and |*R*_*xy*_|, corresponding to the HH, VV, HV, and VH channels. Therefore, the present design targets full‐polarimetric control in the conventional H/V measurement basis, rather than polarization‐insensitive operation for arbitrary incident polarization states. To address this issue, an isotropic reconfigurable metasurface element design can be considered in the future work.

### 2.2 Time‐Modulated Control Circuit Design

To drive the proposed DTMM, we have developed an efficient time‐modulated circuit as shown in Figure 3. The overall DTMM array consists of 20 × 20 elements, with each column of elements connected in parallel. Both ends of every column are further connected in parallel to the V_1_ and V_2_ bus lines, thereby ensuring that all elements across the array operate under the same voltage. An arbitrary waveform generator (AWG) model SDG1062X Plus is employed to generate either unipolar or bipolar voltage pulse waveforms. After passing through a power amplifier (PA) model Aigtek ATA‐4014C, the signals are connected to the V_1_ and V_2_ bus lines, respectively. A unipolar waveform drives the DTMM between co‐polarized full reflection state *S*_*0*_ and either cross‐polarized conversion states *M*_+_ or *M*_*−*_, and is therefore used mainly for amplitude time modulation for both co‐polarized and cross‐polarized channels. A bipolar waveform drives the DTMM between *M*_+_ and *M*_−_, and is therefore used for phase time modulation in the cross‐polarized channel. Notably, the cross‐polarization conversion ratio can be controlled by adjusting the amplification factor of the input voltage, thus allowing RCS and HRRP co‐modulation. For supplementary instructions on the control circuit, please refer to Supporting Information S1. The practical control link is shown in Figure 3b. The output waveforms were monitored by an oscilloscope, and stable periodic and pseudorandom square‐wave modulation up to at least 400 kHz was verified. Since the modulation period at 400 kHz is 2.5 *μ*s, which is much longer than the propagation delay along the bias lines of each PIN diode, the temporal skew caused by line propagation is limited for the demonstrated array. For electrically large apertures, dedicated synchronized feeding designs need to be introduced, such as segmented subarray feeding, distributed driving modules and matched‐length bias lines.

**FIGURE 3:**
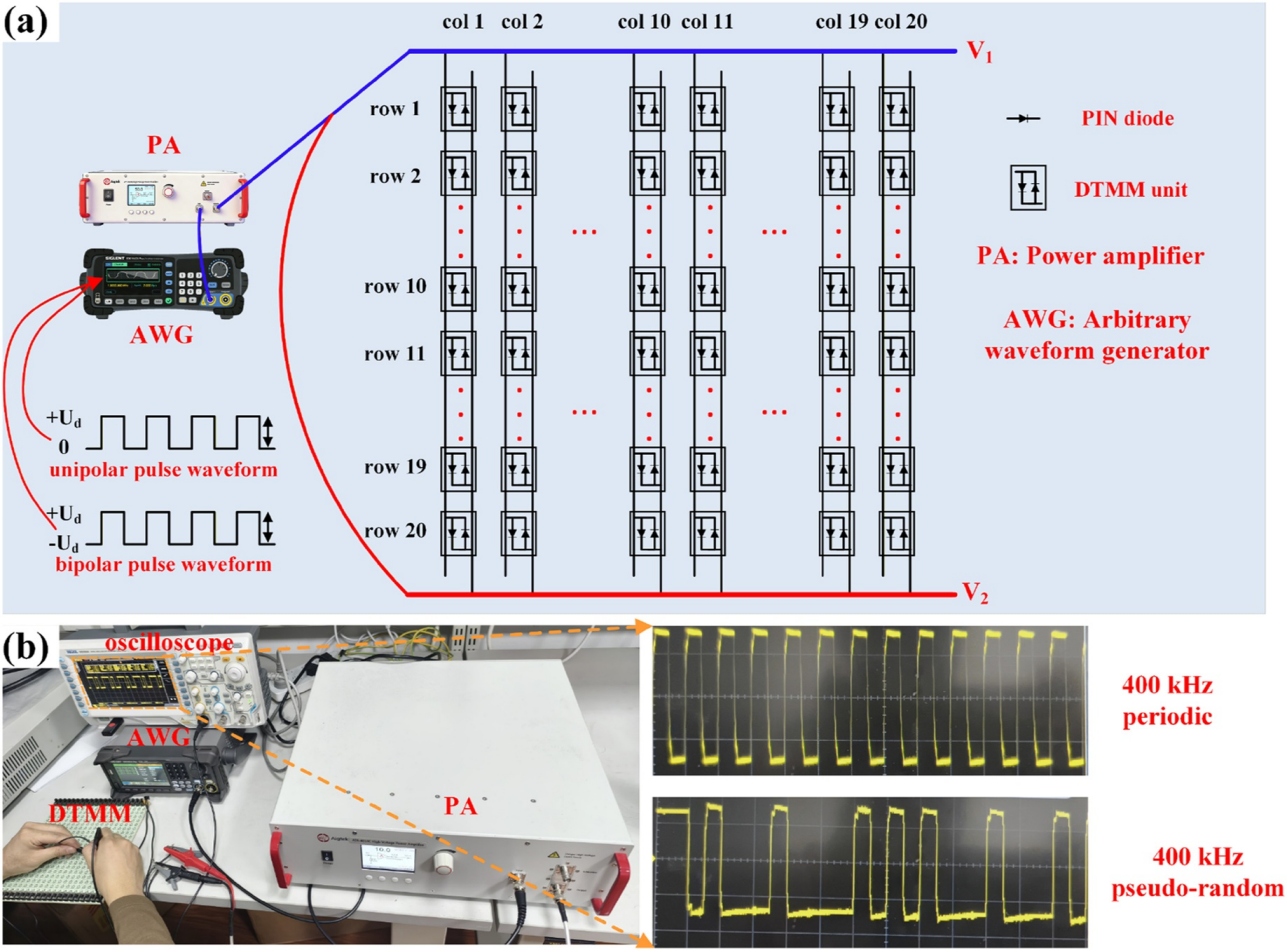
Efficient time‐modulated control circuit design for the proposed DTMM. (a) Schematic diagram and (b) practical control link.

### 2.3 Reflectivity Measurement of DTMM

To conduct the full polarization reflectivity measurements, an arch‐frame test setup is constructed within an anechoic chamber as shown in Figure 4a, and the fabricated DTMM prototype is presented in Figure 4b. By rotating the relative orientation of a pair of transmitting and receiving horn antennas mounted at the center of the arch frame, the co‐polarized and cross‐polarized reflectivity can be measured. Furthermore, rotating the DTMM by 90° allows the polarization of the incident wave to be switched between *x*‐ and *y*‐polarizations. The measured reflectivity results are shown in Figure 4c–h. Under *x*‐polarized incidence, increasing the control voltage from 0 to 1.35 V gradually reduces the co‐polarized reflection amplitude |*R*_*xx*_| by more than 10 dB while enhancing the cross‐polarized reflection amplitude |*R*_*yx*_| by over 30 dB across 7–13.5 GHz, as shown in Figure 4c,d. In addition, Figure 4e shows the cross‐polarized phase differences under two opposite voltage states remain within 180° ± 30° across 7–13 GHz, as the voltage magnitude varies. Correspondingly, Figure 4f–h display the co‐polarized reflectivity |*R*_*yy*_|, cross‐polarized reflectivity |*R*_*xy*_|, and their phase difference under *y*‐polarized incidence at various voltage states. These results demonstrate consistent performance with the *x*‐polarization case, fully confirming the proposed DTMM element's capability for broadband amplitude and phase manipulation across full polarizations. In Supporting Information S1, a comparison table between the proposed DTMM and existing designs is added to highlight its advantages in practical applications.

**FIGURE 4:**
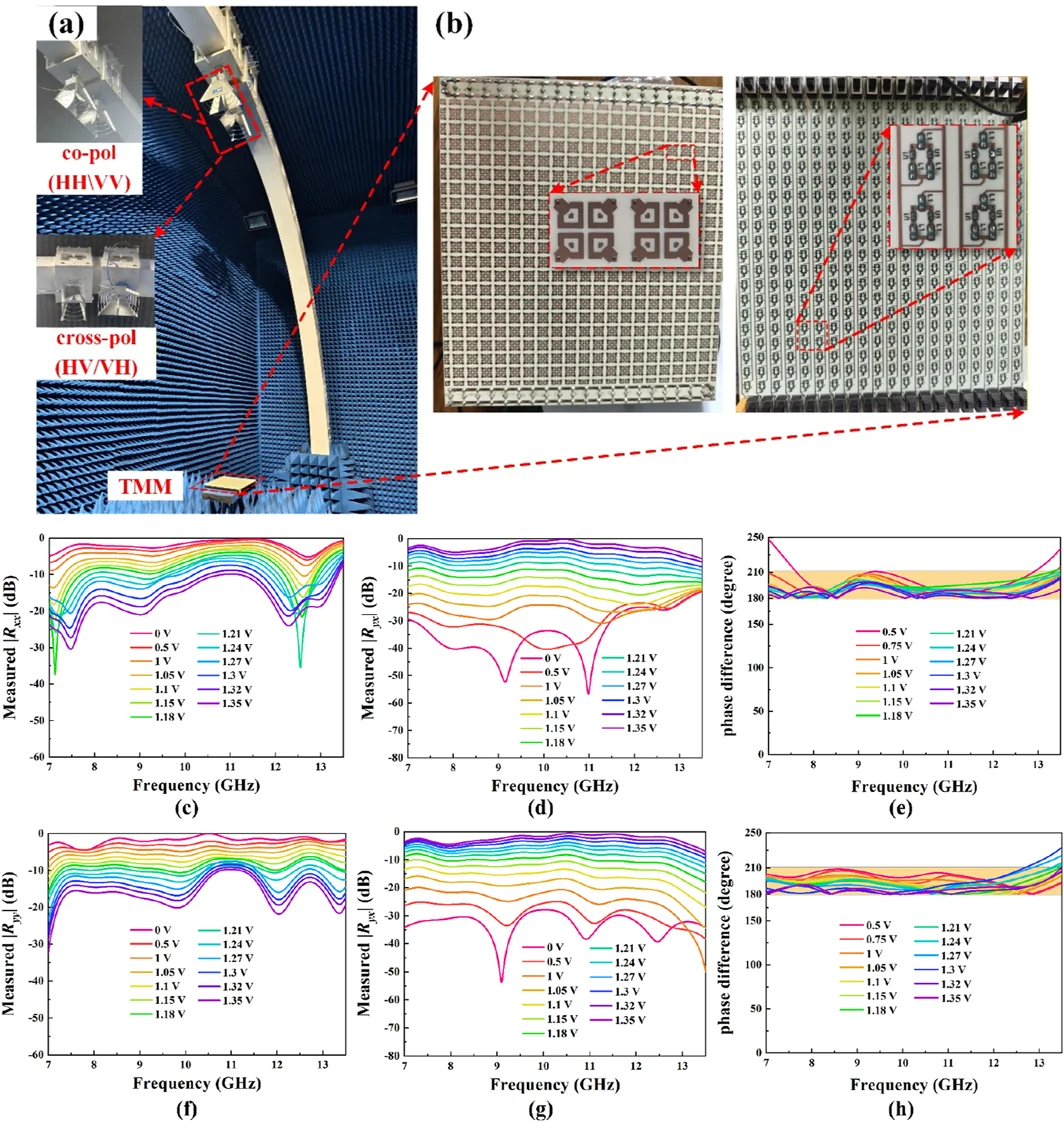
Reflectivity measurements for the proposed DTMM. (a) Test scenario for fully polarized reflectivity. (b) The fabricated prototype. (c, f) The co‐polarized reflectivity for *x*‐ and *y*‐polarized waves. (d, g) The cross‐polarized reflectivity for *x*‐ and *y*‐polarized waves. (e, h) The absolute values of the cross‐polarized reflection phase difference between *M*_+_ and *M*_*−*_ for *x*‐ and *y*‐polarized waves.

## 3 Fully Polarimetric Time‐Modulated Theory

The linear frequency modulation (LFM) waveform is widely used in radar systems due to its long‐distance and high‐resolution detection capability. The expression of the LFM signal is given as

(1) $$S_{t}\left(t\right)=\text{rect}\left(\frac{t}{T_{w}}\right)\exp\left(j2\pi \left(f_{c}t+\frac{1}{2}K_{r}t^{2}\right)\right)$$

(2) $$\text{rect}\left(\frac{t}{T_{w}}\right)=\left\{\begin{array}{ll} 1, & \left|\frac{t}{T_{w}}\right|<0.5\\ 0, & \text{otherwise} \end{array}\right.$$

where $f_{c}$ is the carrier frequency, $T_{w}$ is the pulse width, and $K_{r}=B/T_{w}$ is the chirp rate. The radar wave impinges on the TMM with a time‐varying reflection coefficient Γ(t), and the echo signal $S_{r}\left(t\right)$ is:

(3) $$S_{r}\left(t\right)=S_{t}\left(t\right)\cdot \Gamma \left(t\right)$$

Then, after undergoing mixing and bandpass filtering in the radar receiver, the baseband echo signal can be represented as:

(4) $$R\left(t\right)=\text{rect}\left(\frac{t}{T_{w}}\right)\exp\left(j\pi K_{r}t^{2}\right)\cdot \Gamma \left(t\right)$$

As the proposed DTMM is time‐modulated by switching between two reflection states 1 and 2, the complex reflection coefficients of these two states are defined as $A_{1}e^{j\varphi_{1}}$ and $A_{2}e^{j\varphi_{2}}$. Here, $A_{1}$, $A_{2}$ and $\varphi_{1}$, $\varphi_{2}$ denote the reflection amplitude and phase, respectively. When a periodic coding waveform is applied, its time‐domain expression is given by:

(5) $$\Gamma_{\text{periodic}}\left(t\right)=\left(A_{1}e^{j\phi_{1}}-A_{2}e^{j\phi_{2}}\right)\text{rect}\left(\frac{t}{T_{z}}\right)⊗\sum_{n=-\infty }^{+\infty }\delta \left(t-nT_{m}\right)+A_{2}e^{j\phi_{2}}$$

where $T_{m}$ is the period of the time‐coding sequence and ⊗ denotes the convolution operation. $T_{z}$ and $\alpha =T_{z}/T_{c}$ denotes the code width and duty cycle of reflection state 1, respectively. Then, substituting Equation (5) into Equation (4), the modulated HRRP $I_{\text{periodic}}\left(t\right)$ after matched filtering can be expressed as:

(6) $$I_{\text{periodic}}\left(t\right)=a_{0}+\sum_{n=-N,n\neq 0}^{+N}a_{n}\left(1-\frac{nf_{m}}{B}\right)\text{sinc}\left[K_{r}\left(T_{w}-\left|t\right|\right)\left(t+\frac{nf_{m}}{K_{r}}\right)\right]$$

where $f_{m}=1/T_{m}$ denotes the periodic modulation frequency, and sinc(x)=sin(πx)/(πx). Analysis of Equation (6) reveals that the HRRP after periodic modulation contains multiple discrete sinc false target peaks, where n=±1,±2,…,±N denotes the order of the false target peaks. The amplitude of the *n*th‐order peak after periodic modulation can be expressed as:

(7) $$a_{n}=\left\{\begin{array}{l} \alpha \left(A_{1}e^{j\phi_{1}}-A_{2}e^{j\phi_{2}}\right)+A_{2}e^{j\phi_{2}},n=0\\ \alpha \left(A_{1}e^{j\phi_{1}}-A_{2}e^{j\phi_{2}}\right)\text{sinc}\left(n\alpha \right),n\neq 0 \end{array}\right.$$

where the position of the *n*th‐order false target peak is $d_{n}=ncT_{w}/BT_{m}$, and the distance between adjacent harmonic peaks is $\Delta d=cT_{w}/BT_{m}$.

For a pseudorandom time‐coding sequence of length M, its time‐domain expression is:

(8) $$\Gamma_{\text{random}}\left(t\right)=\left(A_{1}e^{j\phi_{1}}-A_{2}e^{j\phi_{2}}\right)\text{rect}\left(\frac{t}{\tau }\right)⊗\sum_{k=0}^{M-1}r_{k}\delta \left(t-k\tau \right)+A_{2}e^{j\phi_{2}}$$

where $r_{k}$ denotes a randomly reflection state for the *n*th code element, and τ is the code width of the pseudorandom coding sequence. Similarly, substituting Equation (8) into Equation (4), the modulated HRRP $I_{\text{random}}\left(t\right)$ after matched filtering can be expressed as:

(9) $$I_{\text{random}}\left(t\right)=\left(b_{0}+b_{n}\right)\left(1-\frac{\left|t\right|}{T_{w}}\right)\text{sinc}\left[K_{r}t\left(T_{w}-\left|t\right|\right)\right]$$

The amplitude of the *n*th‐order peak after pseudorandom modulation can be expressed as:

(10) $$b_{n}=\left\{\begin{array}{l} \beta \left(A_{1}e^{j\phi_{1}}-A_{2}e^{j\phi_{2}}\right)+A_{2}e^{j\phi_{2}},n=0\\ \tau \left(A_{1}e^{j\phi_{1}}-A_{2}e^{j\phi_{2}}\right){F^{-1}\left[\sum_{k=1}^{M-1}r_{k}\text{sinc}\left(kf\tau \right)\right]|}_{t=nT_{w}/B\tau },n\neq 0 \end{array}\right.$$

where $F^{-1}\left[\cdot \right]$ denotes the inverse Fourier transform, β denotes the duty cycle of reflection state one in the pseudorandom coding sequence. $b_{n,n\neq 0}$ is formed by the linear superposition of continuous harmonic peaks, and its overall amplitude is relatively small. Therefore, the HRRP after random modulation contains only a *0*th‐order peak.

In Section 2, the proposed DTMM possesses the capability of full polarization manipulation, and its complex reflection coefficient Γ(t) can be expressed in matrix form as follows:

(11) $$\Gamma_{a}=\left[\begin{array}{cc} \Gamma_{a}^{xx} & \Gamma_{a}^{yx}\\ \Gamma_{a}^{xy} & \Gamma_{a}^{yy} \end{array}\right]=\left[\begin{array}{cc} A_{a}^{xx}e^{j\varphi_{a}^{xx}} & A_{a}^{yx}e^{j\varphi_{a}^{yx}}\\ A_{a}^{xy}e^{j\varphi_{a}^{xy}} & A_{a}^{yy}e^{j\varphi_{a}^{yy}} \end{array}\right],a=1\;or\;2$$

where the superscript denotes the polarization channel, whereas subscript indicates one of the two reflection states under time modulation.

To achieve jamming against HH and VV polarizations, time modulation of $\Gamma_{a}^{xx}$ and $\Gamma_{a}^{yy}$ is required. When the co‐polarized time‐coding amplitude waveform is periodic as shown in Figure 5a, $A_{1}=1,A_{2}=1-\Delta x_{co},\varphi_{1}=\varphi_{2}$, and α=0.5. Therefore, Equation (7) can be expressed as:

(12) $$a_{n}^{co}=\left\{\begin{array}{l} 1-0.5\Delta x_{co},n=0\\ 0.5\Delta x_{co}\text{sinc}\left(0.5n\right),n\neq 0 \end{array}\right.$$

**FIGURE 5:**
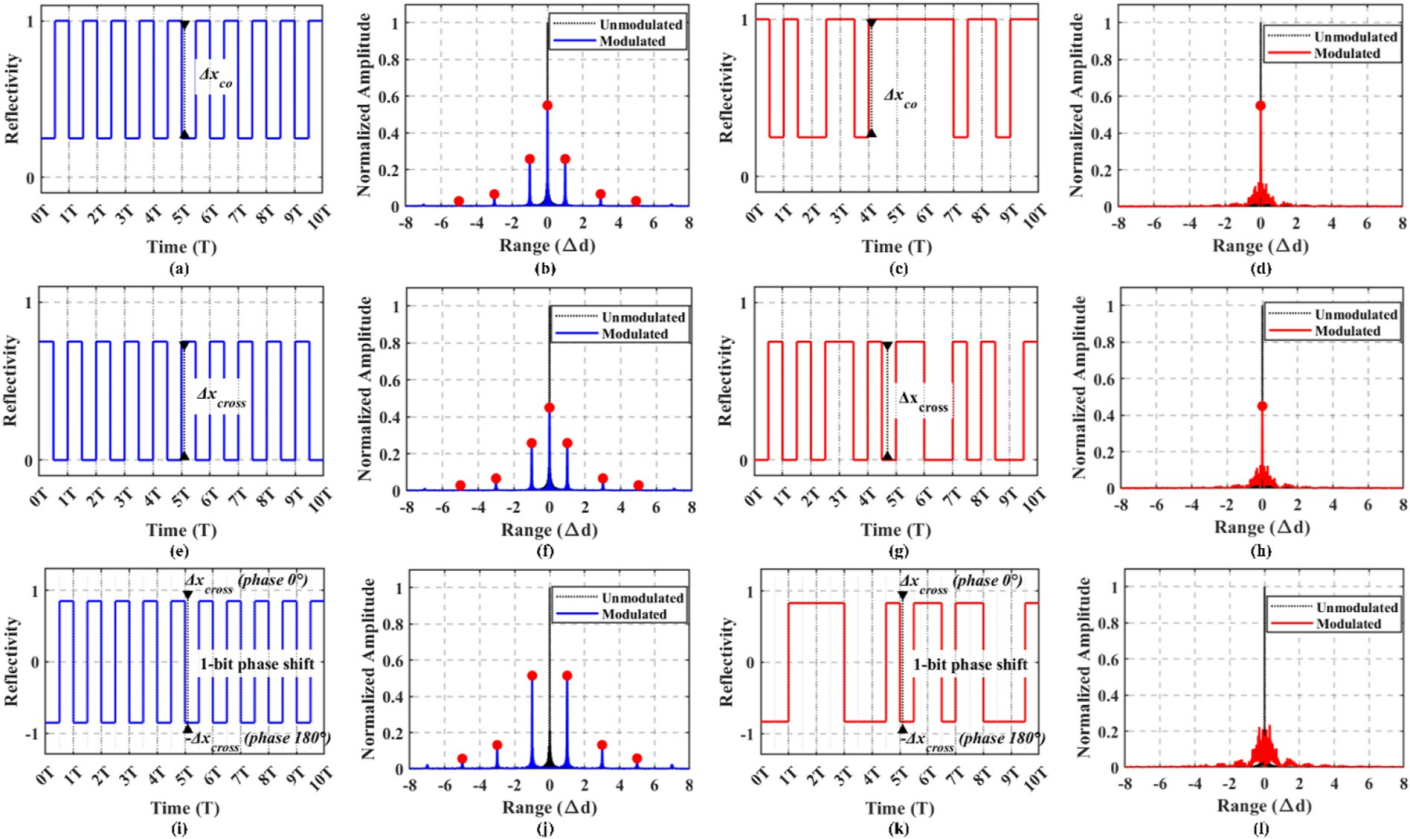
Time code waveforms for different polarizations and the corresponding simulated HRRP modulation results. For co‐polarizations (HH, VV), (a) periodic amplitude time‐coding waveform and (b) HRRP modulation results, (c) pseudorandom amplitude time‐coding waveform and (d) HRRP modulation results. For cross‐polarizations (HV, HV), (e) periodic amplitude time‐coding waveform and (f) HRRP modulation results, (g) pseudorandom amplitude time‐coding waveform and (h) HRRP modulation results, (i) periodic phase time‐coding waveform and (j) HRRP modulation results, (k) pseudorandom phase time‐coding waveform and (l) HRRP modulation results.

It can be observed that the modulated HRRP exhibits a reduction of $0.5\Delta x_{\text{co}}$ in the amplitude of the 0th order peak and generates additional odd order false target peaks compared to the unmodulated HRRP, as illustrated in Figure 5b. Moreover, as $\Delta x_{\text{co}}$ increases, the amplitude of the 0th‐order peak decreases, whereas the amplitudes of the other‐order peaks increase.

When the co‐polarized time‐coding amplitude waveform is pseudorandom as shown in Figure 5c, Equation (8) can be expressed as:

(13) $$b_{n}^{co}=\left\{\begin{array}{l} \left(\beta -1\right)\Delta x_{co}+1,n=0\\ \tau \Delta x_{co}{F^{-1}\left(\sum_{k=1}^{M-1}r_{k}\text{sinc}\left(kf\tau \right)\right)|}_{t=nT_{w}/B\tau },n\neq 0 \end{array}\right.$$

As shown in Figure 5d, the modulated HRRP exhibits a reduction of $\left(1-\beta \right)\Delta x_{\text{co}}$ in the amplitude of the 0th order peak and generates contiguous false target peaks adjacent to it, forming an interference strip. Furthermore, as $\Delta x_{\text{co}}$ increases, the amplitude of the 0th‐order peak decreases, whereas the overall amplitude of the interference strip increases.

To achieve jamming against HV and VH polarizations, time modulation of $\Gamma_{a}^{xy}$ and $\Gamma_{a}^{yx}$ is required. When the cross‐polarized time‐coding amplitude waveform is periodic as shown in Figure 5e, $A_{1}=\Delta x_{cross},A_{2}=0,\varphi_{1}=\varphi_{2}$, and α=0.5. Substituting into Equation (7), the amplitude of each harmonic is given by:

(14) $$a_{n}^{cross}=\left\{\begin{array}{l} 0.5\Delta x_{cross},n=0\\ 0.5\Delta x_{cross}\text{sinc}\left(0.5n\right),n\neq 0 \end{array}\right.$$

A comparison between Equations (12) and (14) reveals that the modulated cross‐polarized HRRP result is similar to its co‐polarized counterpart, except that the amplitude of the 0th‐order peak increases with $\Delta x_{\text{cross}}$, as illustrated in Figure 5f.

When the co‐polarized time‐coding amplitude waveform is pseudorandom as shown in Figure 5g, the amplitude of each harmonic is given by:

(15) $$b_{n}^{\text{cross}}=\left\{\begin{array}{l} \beta \Delta x_{\text{cross}},n=0\\ \tau \Delta x_{\text{cross}}{F^{-1}\left(\sum_{k=1}^{M-1}r_{k}\text{sinc}\left(kf\tau \right)\right)|}_{t=nT_{w}/B\tau },n\neq 0 \end{array}\right.$$

In contrast to the co‐polarized result given by Equation (13), as $\Delta x_{\text{cross}}$ increases, the amplitude of the 0th‐order peak becomes larger, and the overall amplitude of the interference strip also increases, as illustrated in Figure 5h.

Due to the additional 1‐bit phase shift capability under cross‐polarization of the proposed DTMM unit, phase time modulation can also be applied to the HV and VH polarizations. When the co‐polarized time‐coding phase waveform is periodic as shown in Figure 5i, $A_{1}=A_{2}=\Delta x_{cross},\left|\varphi_{1}-\varphi_{2}\right|=\pi$, and α=0.5. Therefore, the amplitude of each harmonic can be further expressed as:

(16) $$a_{n}^{\text{cross}}=\left\{\begin{array}{l} 0,n=0\\ \Delta x_{\text{cross}}\text{sinc}\left(0.5n\right),n\neq 0 \end{array}\right.$$

When the co‐polarized time‐coding phase waveform is pseudorandom as shown in Figure 5k, the amplitude of each harmonic can be further expressed as:

(17) $$b_{n}^{\text{cross}}=\left\{\begin{array}{l} \left(2\beta -1\right)\Delta x_{\text{cross}},n=0\\ 2\tau \Delta x_{\text{cross}}{F^{-1}\left(\sum_{k=1}^{M-1}r_{k}\text{sinc}\left(kf\tau \right)\right)|}_{t=nT_{w}/B\tau },n\neq 0 \end{array}\right.$$

With the introduction of phase modulation, compared to pure amplitude modulation, the amplitude of the 0th‐order peak on the modulated HRRP approaches 0, whereas the tunable range of amplitudes for the other harmonic peaks is significantly expanded, as shown in Figure 5j,l. This not only enables the modulation of false targets but also conceals the central 0th‐order peak. According to Equations ((12), (13), (14), (15), (16), (17)), more flexible manipulation of harmonic peaks can be achieved by controlling the amplitude $\Delta x_{\text{co}}$ and $\Delta x_{\text{cross}}$ between the two states in the time modulation.

It should be noted that the objective of the proposed DTMM is not limited to ideal target invisibility. In practical ECM scenarios, RCS and HRRP co‐modulation can be used for target weakening, target camouflage (background‐feature matching), false‐target generation, and range‐domain masking. Therefore, complete suppression of all co‐polarized scattering components is not a necessary condition for the proposed method. Instead, the DTMM aims to dynamically redistribute and modulate the scattering energy between two orthogonal polarized channels, with a certain tolerance for polarization isolation.

## 4 Result and Analysis of RCS and HRRP Co‐Modulation

Building upon the DTMM and its corresponding polarimetric time‐modulation theory, further experimental validation of its co‐manipulation capability for both RCS and HRRP is carried out. The experimental setup, as illustrated in Figure 6, is constructed in an anechoic chamber. A signal generator (Rohde & Schwarz SMW200A) is connected to the transmitting horn antenna, whereas a spectrum analyzer (Rohde & Schwarz FSW43) is linked to the receiving horn antenna, jointly simulating a radar system for transmitting and receiving LFM signals to detect targets. Different polarization channels are emulated by rotating the apertures of the horn antennas. The fabricated DTMM is positioned on a white foam platform at a distance of approximately 3 m from the horn antennas. The time‐coding sequence input to the DTMM is generated by the controller depicted in Figure 3. When the signal generator outputs a unipolar voltage signal ranging from 0 to $+U_{d}$, the co‐polarized amplitude of the DTMM undergoes time modulation between 1 and $1-\Delta x_{co}$, whereas its cross‐polarized amplitude is time‐modulated between 0 and $\Delta x_{\text{cross}}$. When a bipolar voltage signal ranging from $-U_{d}$ to $+U_{d}$ is output, its cross‐polarization amplitude is time‐modulated between $-\Delta x_{\text{cross}}$ and $+\Delta x_{\text{cross}}$, namely, phase modulation. Additionally, as shown in Figure 4, both $\Delta x_{\text{co}}$ and $\Delta x_{\text{cross}}$ increase as $U_{d}$ becomes larger. The transmitted signal, after being reflected by the DTMM, is received as a modulated echo signal, producing the intended modulation effects in the resulting HRRP. The parameters of the transmitted radar signals are: center frequency $f_{c}=10\;\text{GHz}$, bandwidth B=300MHz and pulse width $T_{w}=1.024\;\text{ms}$.

**FIGURE 6:**
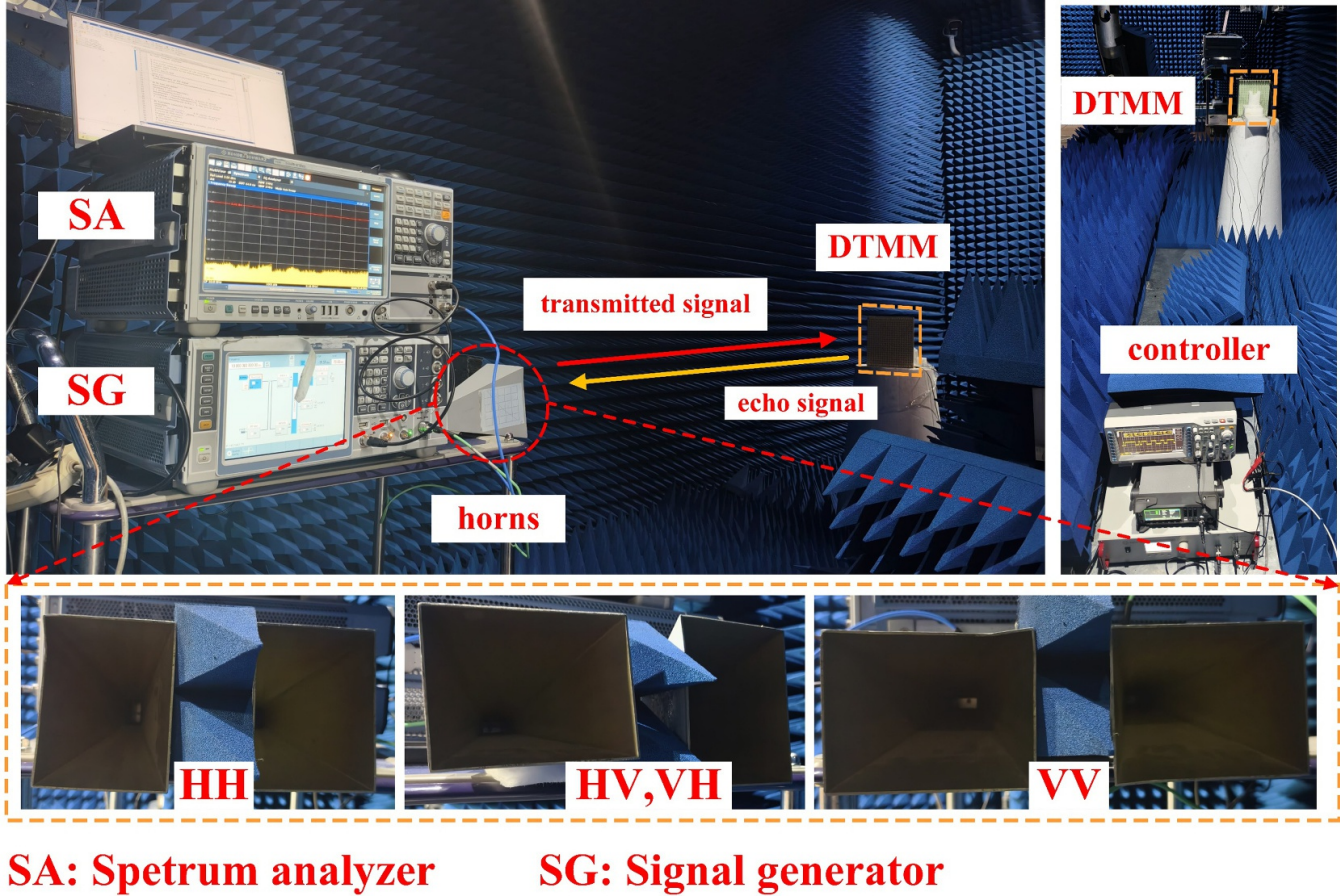
Experimental scenario of RCS and HRRP co‐manipulation based on the DTMM.

Figure 7 shows the measured results of full polarization HRRP manipulation using the DTMM under varying voltage difference $U_{d}$ without time modulation, and the metasurface is located at approximately 3 m in the HRRP, which is in good agreement with the actual distance. In Figure 7a,b, as $U_{d}$ increases, the co‐polarized reflection intensity of the metasurface gradually weakens, corresponding to a decreasing $\Delta x_{co}$, which is consistent with the trend of the reflectivity results shown in Figure 4c,f. Conversely, in Figure 7c, the cross‐polarized reflection intensity grows with increasing $U_{d}$, meaning $\Delta x_{cross}$ becomes larger, which also agrees with the reflectivity results in Figure 4d,g. Thus, by adjusting the voltage difference $U_{d}$, dynamical RCS control for a real target under full polarizations can be achieved, enabling stealth or camouflage effects.

**FIGURE 7:**
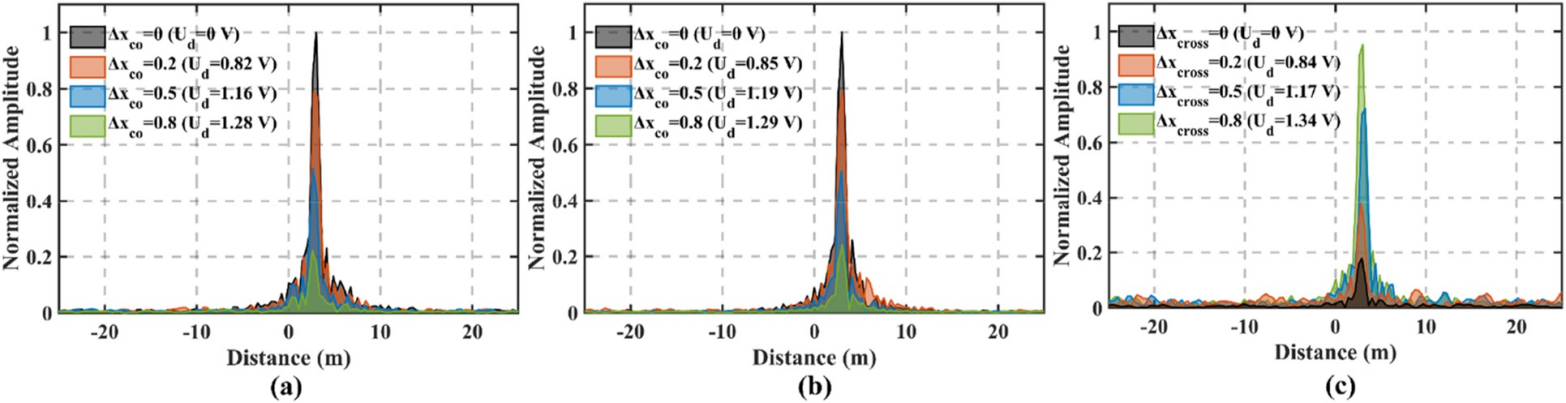
Experimental HRRP results for (a) HH, (b) VV, (c) HV/VH polarizations using the DTMM at varying voltage difference $U_{d}$ without time modulation.

Figure 8 presents the measured full‐polarimetric HRRP responses under periodic time modulation. The results are arranged to demonstrate two independent control capabilities. In the first column, the modulation frequency is fixed at $f_{m}=50\text{kHz}$ whereas the modulation depth is varied. The control voltages corresponding to different modulation depths in each polarization channel are obtained through a mapping lookup table based on the experimental data collected in Figure 7. (See Supporting Information S1 for details) In the co‐polarized HH and VV channels, increasing $\Delta x_{co}$ gradually suppresses the central target peak and enhances the false targets. This trend agrees with Equation (12), indicating that the modulation depth directly controls the energy transfer from the real target to the harmonic false targets. In the cross‐polarized HV/VH channels, increasing $\Delta x_{cross}$ enhances the cross‐polarized jamming components, which is consistent with Equation (14). When joint amplitude–phase modulation is applied, the central target peak is significantly suppressed because the two opposite cross‐polarized states contribute with an approximately 180° phase difference, as predicted by Equation (16). This verifies the self‐blanking capability of the proposed DTMM. In the second column of Figure 8, the modulation depth is fixed at $\Delta x_{\text{co}}=0.8$ for HH/VV and $\Delta x_{\text{cross}}=0.8$ for HV/VH, whereas the modulation frequency *f*_*m*_ is swept from 25 to 100 kHz. According to Equation (6), the false‐target spacing is determined by $\Delta d=cT_{w}/BT_{m}$. Therefore, increasing $f_{m}=1/T_{m}$ enlarges the range separation between adjacent harmonic peaks. The measured results show that the false‐target spacing increases from approximately 25–100 m, confirming that the generated false targets can be placed at programmable range positions by controlling the temporal modulation frequency. To verify the broadband time‐modulated performance of DTMM, the HRRP modulation results and analysis at the edge carrier frequency are fully discussed in Supporting Information S1.

**FIGURE 8:**
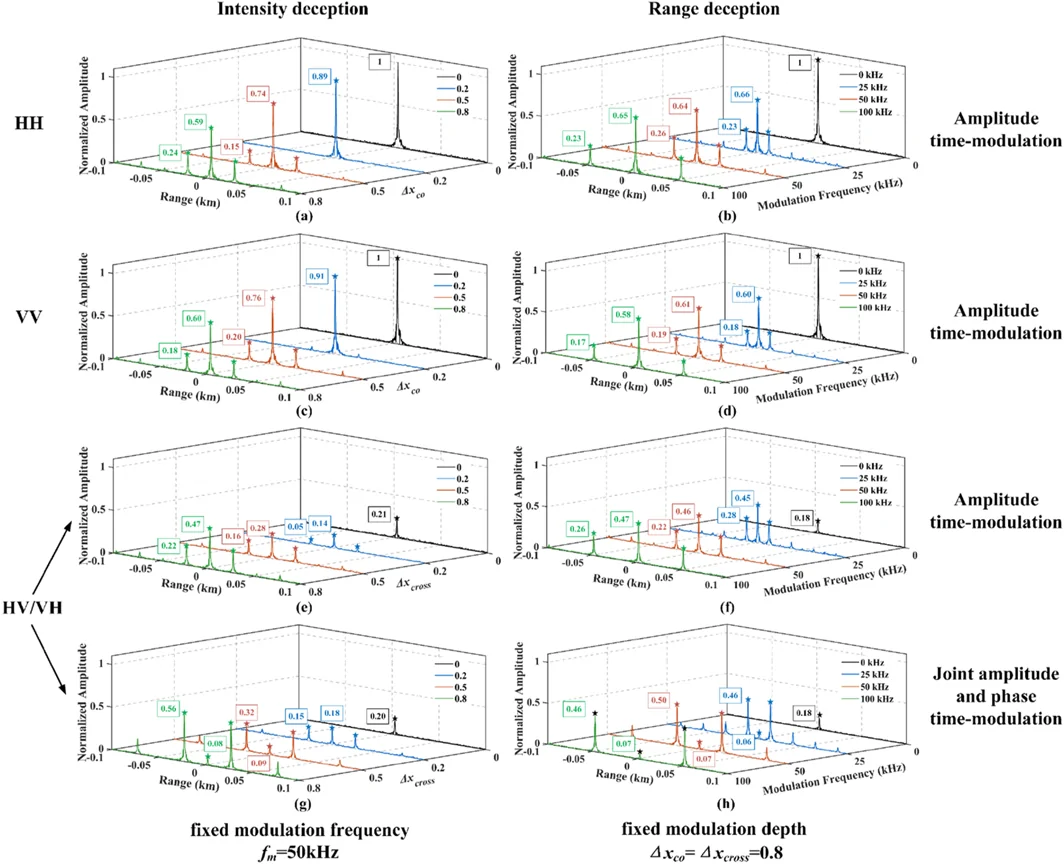
Experimental results of full polarization HRRP manipulation results based on periodic time modulation of DTMM. HH polarization under amplitude time modulation with varying modulation (a) depth $\Delta x_{\text{co}}$ and (b) frequency *f*_*m*_. VV polarization under amplitude time modulation with varying modulation (c) depth $\Delta x_{\text{co}}$ and (d) frequency *f*_*m*_. HV/VH polarization under amplitude time modulation with varying modulation (e) depth $\Delta x_{\text{cross}}$ and (f) frequency *f*_*m*_. HV/VH polarization under joint amplitude and phase time modulation with varying modulation (g) depth $\Delta x_{\text{cross}}$ and (h) frequency *f*_*m*_.

To further enrich the HRRP jamming patterns of the DTMM, Figure 9 shows the measured full‐polarimetric HRRP responses under pseudorandom time modulation. Different from periodic modulation, pseudorandom coding does not concentrate the modulated energy into several discrete harmonic false targets. Instead, the random temporal sequence produces a superposition of continuous harmonic components, forming an interference strip around the real target. In the first column, the modulation frequency is fixed at $f_{m}=50kHz$ and the modulation depth is varied. For the co‐polarized HH and VV channels, increasing $\Delta x_{co}$ reduces the real‐target peak and increases the overall amplitude of the interference strip, consistent with Equation (13). For the cross‐polarized HV/VH channels, increasing $\Delta x_{cross}$ strengthens the generated interference components, consistent with Equation (15). When joint amplitude–phase modulation is introduced, the central peak is further suppressed, demonstrating that the phase degree of freedom can be used to combine range‐domain masking with target self‐blanking. In the second column, the modulation depth is fixed at $\Delta x_{co}=0.8$ for HH/VV and $\Delta x_{cross}=0.8$ for HV/VH, while the modulation frequency *f*_*m*_ is varied. As the modulation frequency increases, the interference strip becomes broader in the range domain, while its average amplitude slightly decreases because the modulated energy is distributed over a wider range interval. This result confirms that pseudorandom time modulation provides a programmable range‐domain masking capability, complementary to the discrete false‐target generation achieved by periodic modulation. In Supporting Information S1, a comprehensive comparison with the existing ECM techniques based on PMs is conducted, further demonstrating the progressiveness of this work.

**FIGURE 9:**
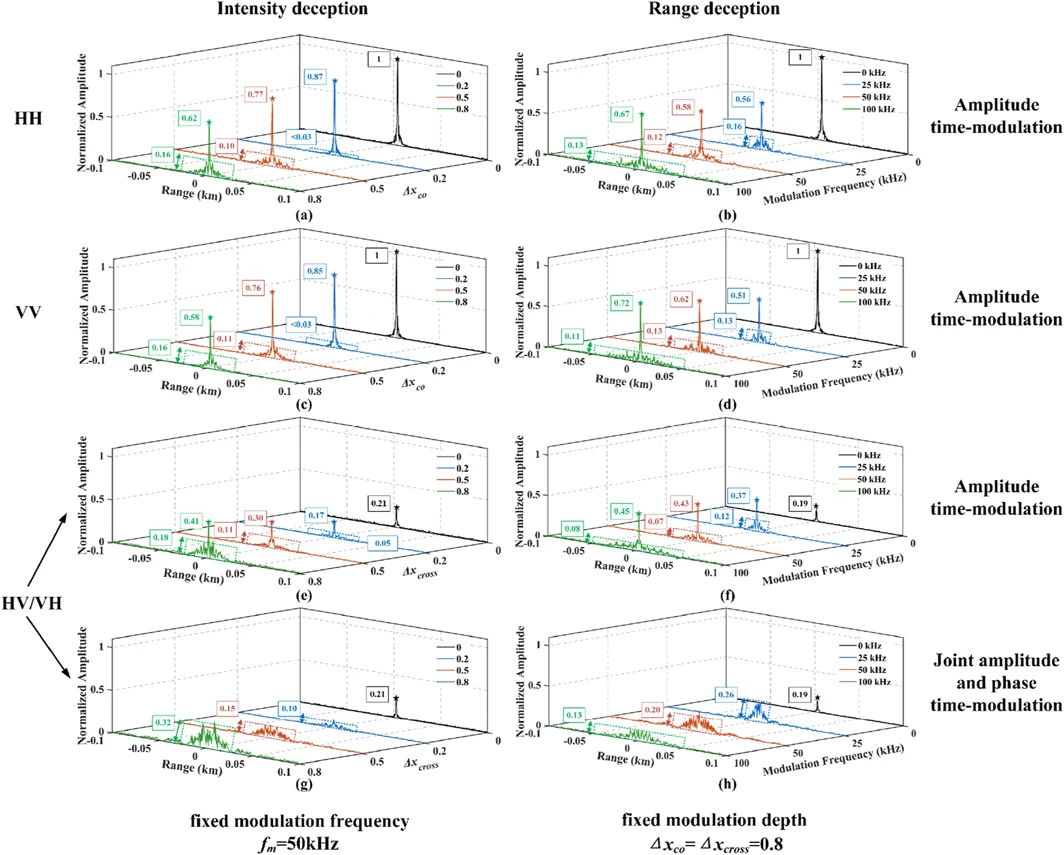
Experimental results of full polarization HRRP manipulation results based on pseudorandom time modulation of DTMM. HH polarization under amplitude time modulation with varying modulation (a) depth $\Delta x_{\text{co}}$ and (b) frequency *f*_*m*_. VV polarization under amplitude time modulation with varying modulation (c) depth $\Delta x_{\text{co}}$ and (d) frequency *f*_*m*_. HV/VH polarization under amplitude time modulation with varying modulation (e) depth $\Delta x_{\text{cross}}$ and (f) frequency *f*_*m*_. HV/VH polarization under joint amplitude and phase time modulation with varying modulation (g) depth $\Delta x_{\text{cross}}$ and (h) frequency *f*_*m*_.

## 5 Conclusion

In this work, we have proposed and experimentally validated a broadband DTMM, capable of full polarimetric RCS and HRRP co‐modulation. The designed element exhibits reconfigurable polarization conversion for *x*/*y* polarizations from 7 to 13.5 GHz, enabling amplitude control across all four polarization quadrants. By integrating two anti‐parallel PIN diodes to manipulate the direction of the induced surface current, it can emulate a 90° rotation of a passive polarization conversion unit, thereby achieving broadband, stable 1‐bit phase shift after orthogonal polarization conversion without the need for complicated design. Based on this DTMM, a time‐modulation theory for the co‐manipulation of full polarization RCS and HRRP is proposed. Experimental validation of its jamming capability is conducted in an anechoic chamber using the fabricated prototype. The measured results demonstrate that periodic and pseudorandom time modulations generate symmetrically distributed odd‐order false targets and interference strips around the real target, respectively. The additional incorporation of phase modulation further facilitates the blanking of the real target in the cross‐polarize domain. By controlling the modulation depth and frequency, precise adjustment of the amplitude and range of the generated radar signatures is achieved, showcasing its flexible jamming capability. This work expands the application scenarios of metasurfaces in the field of ECM.

## Author Contributions

**Hantao Xu:** conceptualization, investigation, methodology, data curation, software, validation, writing – original draft, writing – review and editing, visualization. **Jinxing Guo:** methodology, validation. **Xinyang Mao:** validation, formal analysis. **Shijian Yu:** methodology, formal analysis. **Yihao Xu:** validation, investigation. **Peng You:** writing – review and editing, supervision. **Bin Zheng:** supervision, writing – review and editing. **Dongfang Guan:** conceptualization, investigation, writing – review and editing, funding acquisition, supervision. **Zhen Liu:** supervision, resources, project administration, funding acquisition, writing – review and editing. **Yongxiang Liu:** resources, project administration, writing – review and editing, funding acquisition.

## Conflicts of Interest

The authors declare no conflicts of interest.

## Supporting information


Supporting Information S1

## Data Availability

The data that support the findings of this study are available from the corresponding author upon reasonable request.
