# Supplementary material for: Fully Polarimetric Radar Cross‐Section and High‐Resolution Range Profile Co‐Modulation via Diversely Time‐Modulated Metasurface
Source: Nanophotonics. 2026 Jul 14;15(14):e70205. doi: 10.1002/nap2.70205 (PMC13642073; doi:10.1002/nap2.70205)
Supplement: Supplementary file 1 [file NAP2-15-e70205-s001.pdf]

Supporting Information:

Fully polarimetric RCS and HRRP co-modulation based on diversely time-modulated metasurface

Hantao Xu[1], *Jinxing Guo*[1], *Xinyang Mao*[1], *Shijian Yu*[1], *Yihao Xu*[1], *Peng You*[1], *Bin Zheng*[2], *Dongfang Guan*[1]*, *Zhen Liu*[1]*, *and Yongxiang Liu*[1]

[1]College of Electronic Science and Technology, National University of Defense Technology, Changsha, 410005, China

*E-mail: gdfguandongfang@163.com (*Dongfang Guan*); zhen_liu@nudt.edu.cn (*Zhen Liu*)

[2]Hangzhou Global Scientific and Technological Innovation Center, Zhejiang University, Hangzhou, 311200, China

Contents

S1. The absolute reflection phase and relative phase difference of the DTMM unit in separate panels 1

S2. Simulated results of DTMM unit in polarization conversion state under different oblique incidence angles 2

S3. Simulated surface current distribution results under two different polarization conversion states 3

S4. The evolution process of DTMM unit shape and its relationship with operating bandwidth 5

S5. The schematic diagram of DTMM control circuit 6

S6. Performance comparisons between the proposed DTMM and existing designs 7

S7. Corresponding relationship between modulation depth and control voltage in co-modulation of RCS and HRRP 8

S8. Experimental HRRP results based on the DTMM at edge frequencies 9

S9. Comparisons of different RCS and HRRP modulation techniques based on programmable metasurfaces 10

S1. The absolute reflection phase and relative phase difference of the DTMM unit in separate panels

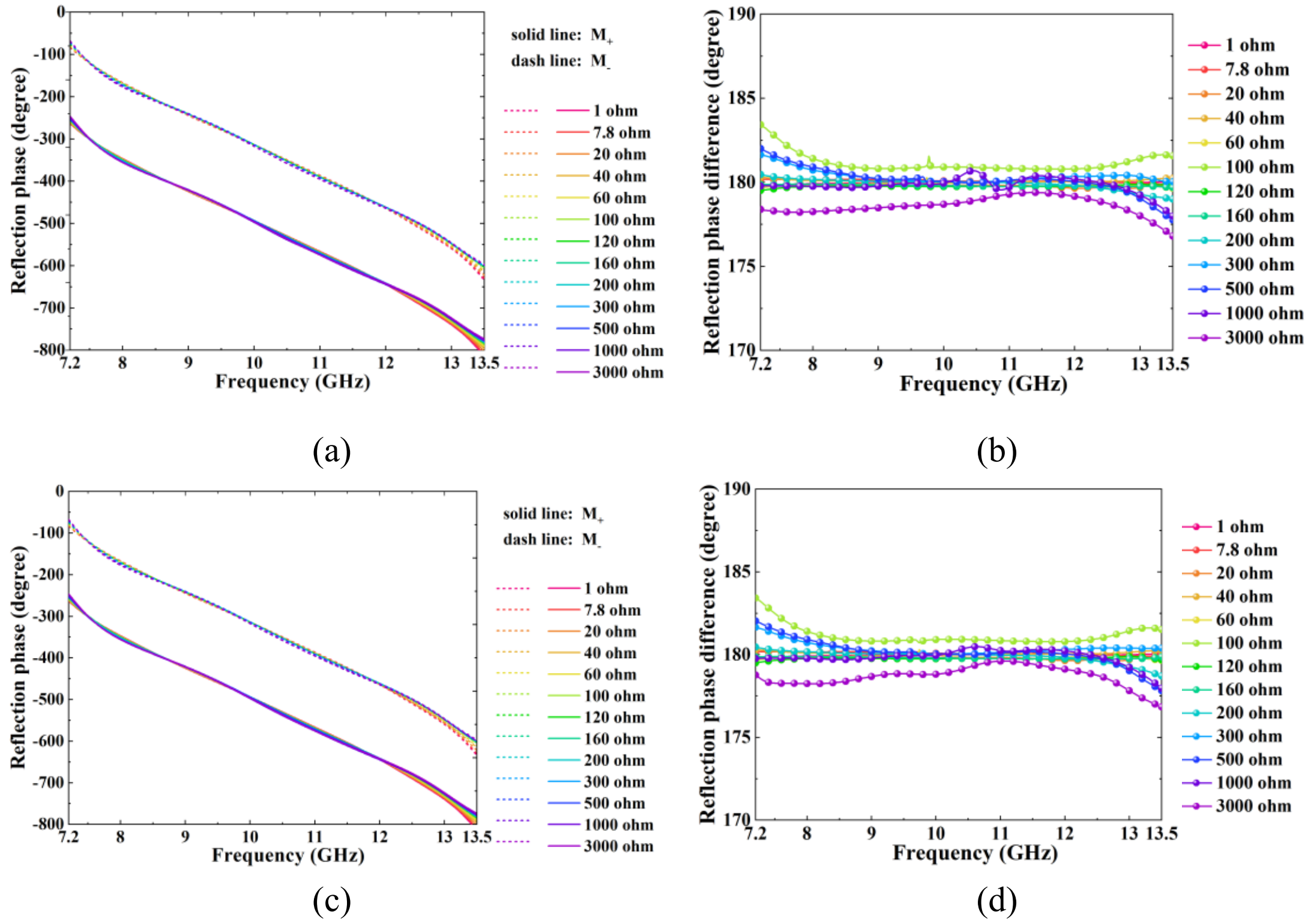

Figure S1. Phase switching under *x*-polarized incidence: (a) cross-polarized reflection phases of M_+ and M_-, and (b) their phase difference. Phase switching under *y*-polarized incidence: (c) cross-polarized reflection phases of M_+ and M_-, and (d) their phase difference.

According to Figure S1, the cross-polarized reflection phase of DTMM cells under two reverse bias states M_+ and M_- remains basically unchanged with the variation of diode resistance R_d. The cross-polarized reflection phase of the M_+ state has excellent parallelism with the M_- state over a wide frequency range, and the phase difference between the two reflection states remains stable between 180°±5° within the operating frequency band of 7.2-13.5GHz. In addition, the phase modulation performance remains consistent under *x*- and *y*-polarized incidence.

S2. Simulated results of DTMM unit in polarization conversion state under different oblique incidence angles

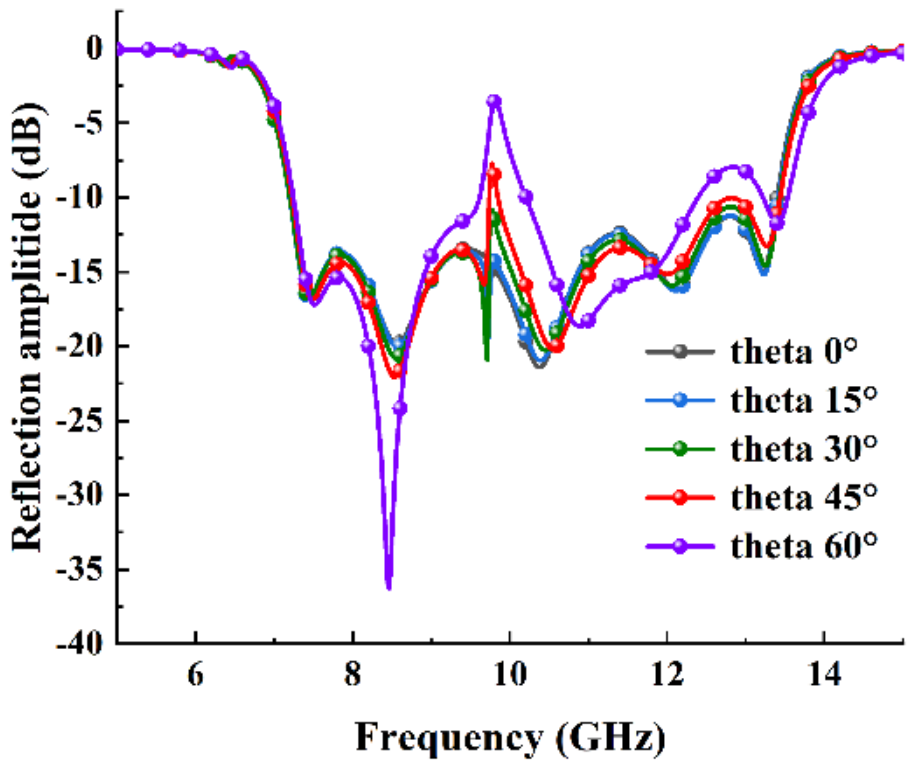

(a)

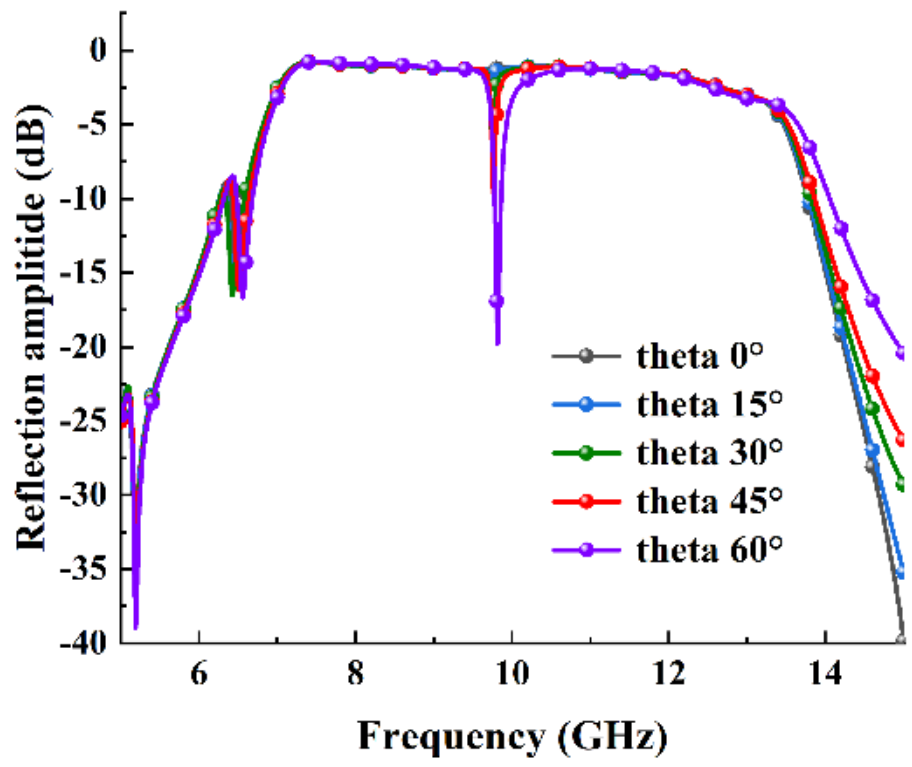

(b)

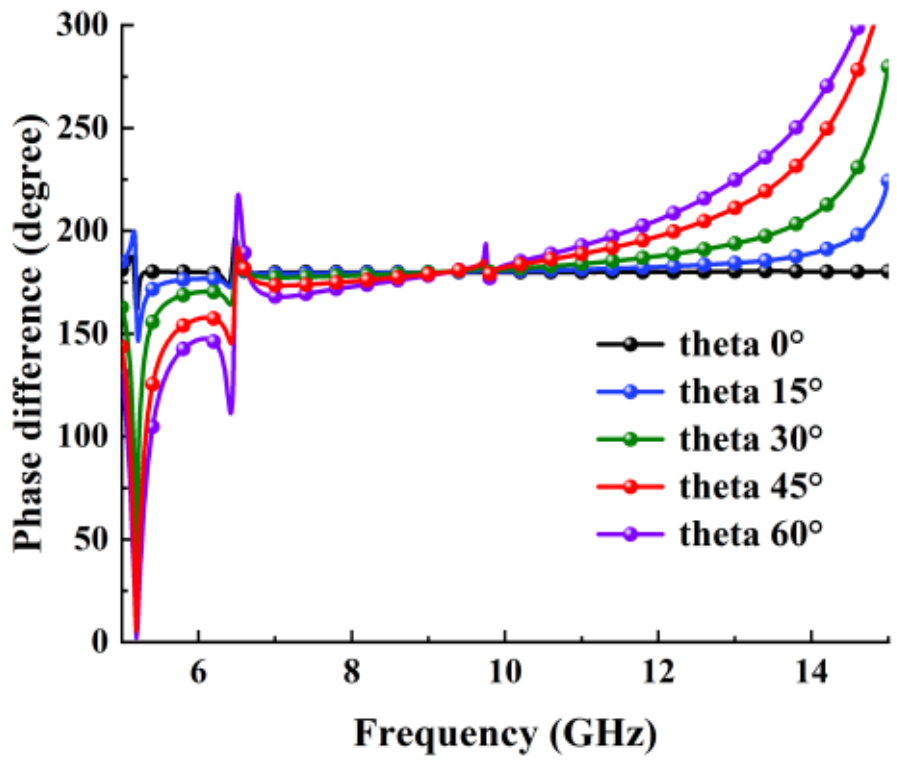

(c)

Figure S2. Simulated reflection responses of the proposed DTMM unit at the cross polarized state under oblique incidence: (a) co-polarized reflection magnitude $|R_{xx}|$, (b) cross-polarized reflection magnitude $|R_{yx}|$ and (c) cross-polarized phase difference $|\varphi_{xx}|$ between M_+ and M_-.

The simulated results of DTMM unit in cross polarization conversion state under x-polarized oblique incidence are shown in Figure S3. Among them, Figures S2(a)-(b) represent the co-polarized reflection amplitude $|R_{xx}|$, cross-polarized reflection magnitude $|R_{yx}|$ and (c) cross-polarized phase difference $|\varphi_{xx}|$ between mode 1 and mode 2, respectively. When the oblique incidence angle is less than or equal to 45°, $|R_{xx}|$ is always less than -10 dB from 7.2 GHz to 13 GHz, $|R_{yx}|$ is greater than -2dB, and $|\varphi_{xx}|$ is within 180°±20°. However, when the angle increases to 60°, the amplitude and phase responses of the DTMM unit deteriorate. Overall, the proposed DTMM unit still exhibits a certain degree of insensitivity to oblique incidence angles.

S3. Simulated surface current distribution results under two different polarization conversion states

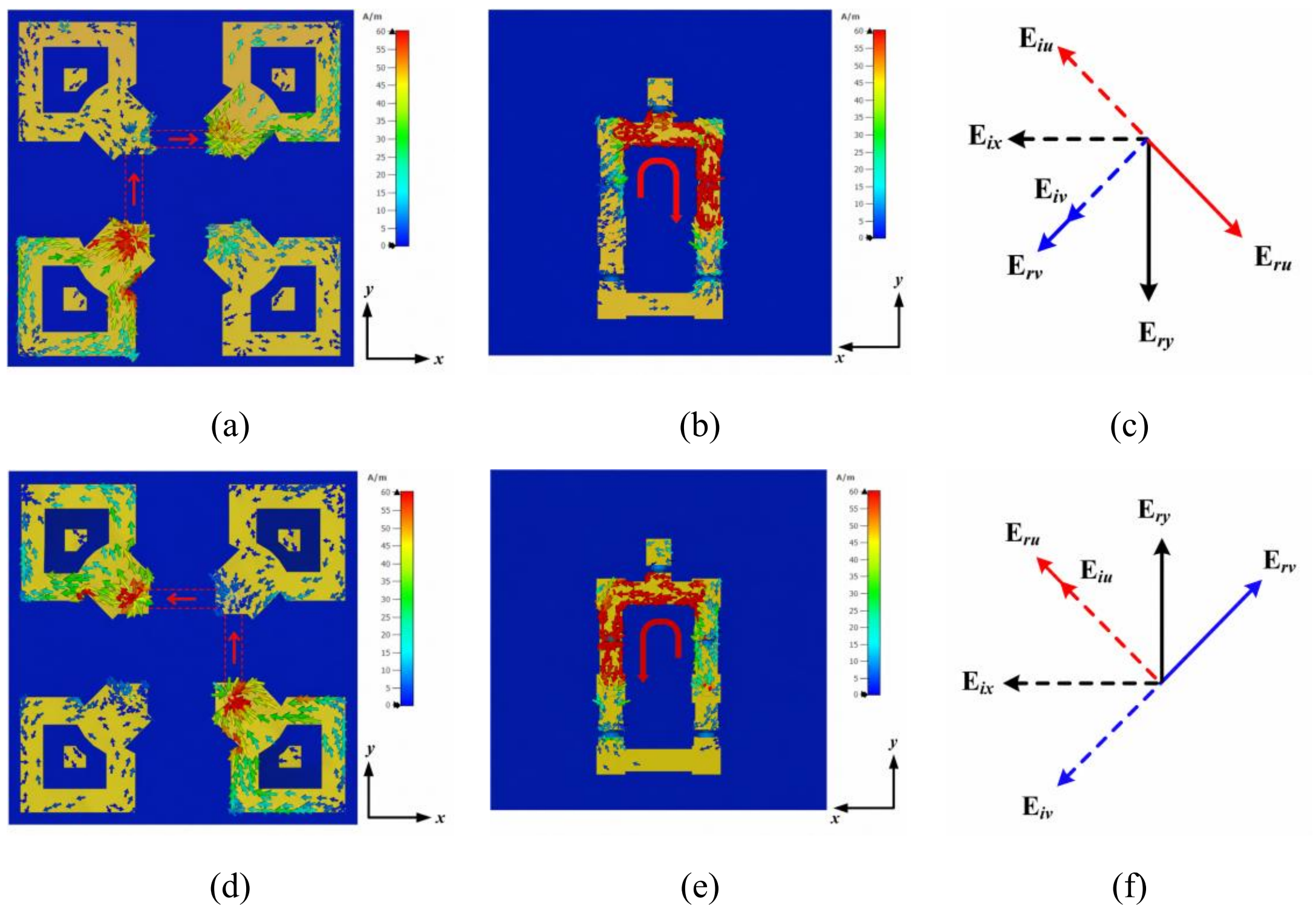

Figure S3. Simulated surface current distributions of the DTMM unit on the top metal patches at (a) M_+ and (d) M_- polarization conversion states, and on the bottom metal strips at (b) M_+ and (e) M_- polarization conversion states. The polarization decomposition diagrams in (c) M_+ and (f) M_- at 10 GHz.

To further clarify the physical mechanism of the reconfigurable polarization conversion, the simulated surface-current distributions of the DTMM unit at 10 GHz are presented in Figure S3. Figures S3(a) and S3(d) show the current distributions on the top metallic patches under M_+ and M_-, respectively, while Figures S3(b) and S3(e) show the corresponding current distributions on the bottom metallic strips. The polarization-decomposition diagrams for the two modes are given in Figures S3(c) and S3(f).

Under M_+ state, as shown in Figures S3(a) and S3(b), the anti-parallel PIN-diode configuration selects one conducting branch, so that the induced current is mainly guided along a specific diagonal path of the unit cell. Strong surface currents are observed on one pair of diagonally arranged quasi-dart-shaped patches, whereas the other pair of patches is weakly coupled or effectively isolated from the dominant current path. This asymmetric current distribution indicates that the unit exhibits distinct electromagnetic responses along two orthogonal diagonal axes. For an incident x-polarized wave, the electric field can be decomposed into two orthogonal components along these diagonal directions as shown in Figure S3(c). The component parallel to the dominant current path experiences a strong electric

response, while the orthogonal component experiences a different effective boundary response. The phase contrast between these two reflected components enables their recombination into an orthogonally polarized reflected wave, thereby realizing linear polarization conversion.

When the bias polarity is reversed, the DTMM unit switches to M_- state. As shown in Figures S3(d) and S3(e), the conducting branch of the anti-parallel PIN-diode pair is exchanged, and the induced current path is correspondingly rotated or reversed with respect to that in M_+. The surface-current distribution in M_- can therefore be regarded as the counterpart of M_+ with an approximately reverse $90°$-rotated current pattern. This reconfigured current path leads to the opposite polarization-rotation behavior of the reflected field, as illustrated by the polarization-decomposition diagram in Figure S3(f). DTMM in M_+ and M_- are similar to two passive polarization conversion metasurfaces that are mutually 90° rotationally symmetric. Consequently, the cross-polarized reflected fields generated in M_+ and M_- have comparable amplitudes but opposite phase polarities, corresponding to an approximately $180°$ phase difference between the two cross-polarization conversion states. This broadband phase difference design does not require complex unit structure optimization, making it relatively simple to implement.

S4. The evolution process of DTMM unit shape and its relationship with operating bandwidth

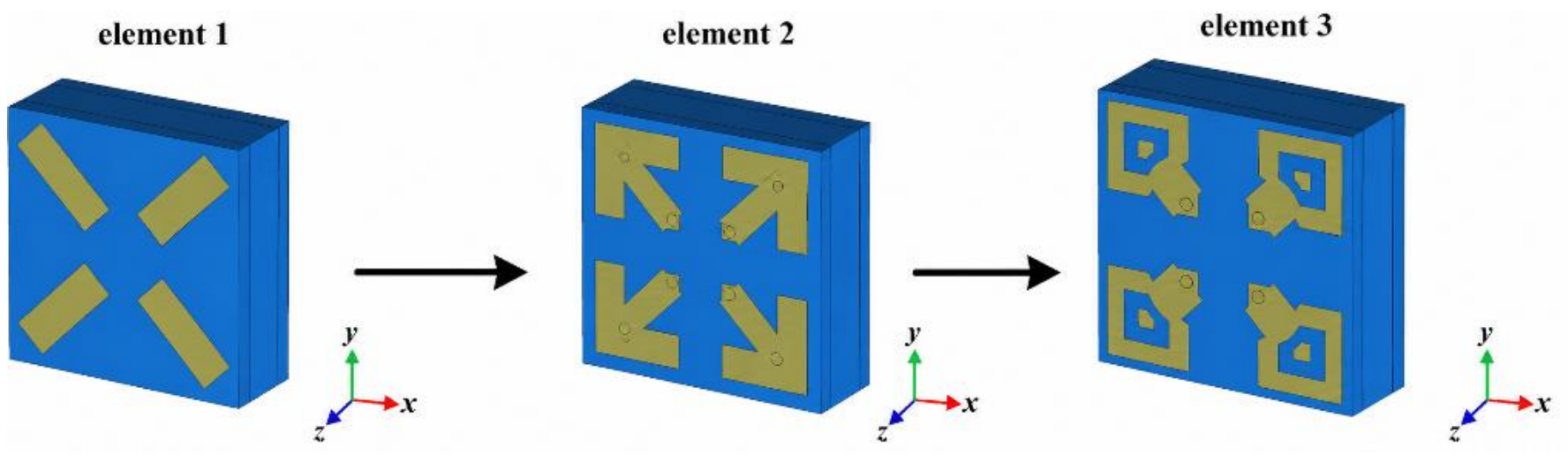

(a)

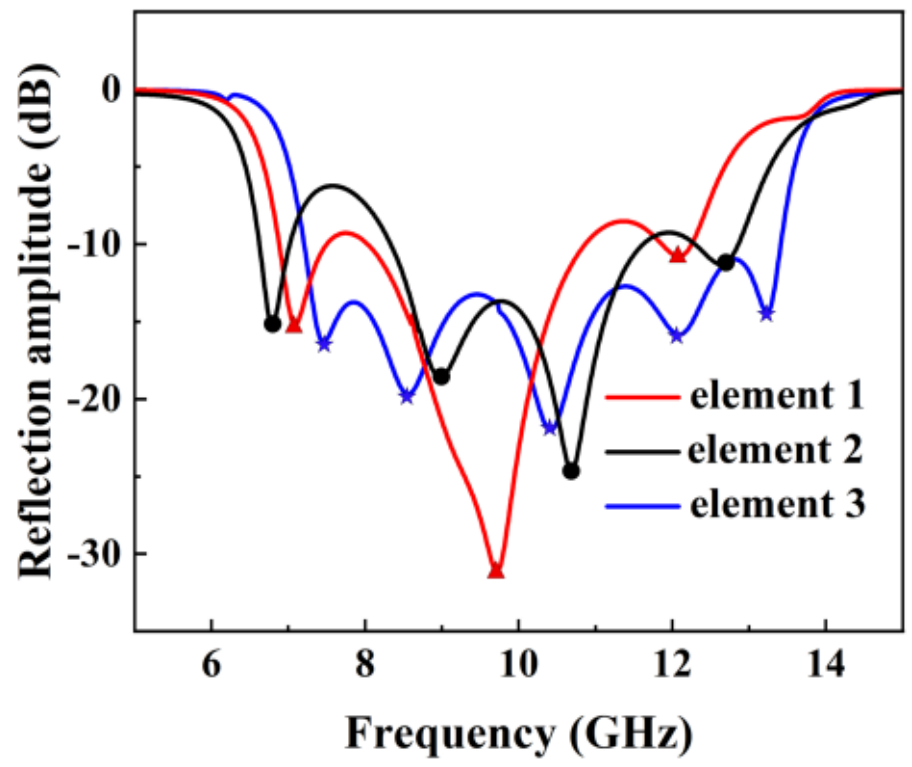

(b)

Figure S4. (a) The evolution process of the proposed DTMM unit shape. (b) The simulated co-polarized reflection amplitude $|R_{xx}|$ at the polarization conversion state. (Red triangle, black circle and blue pentagram denote the resonant points of element 1, element 2 and element 3, respectively)

First, due to the dual-polarization characteristics of the unit cell, its upper patch geometry must exhibit symmetry along both the x-axis and y-axis. Secondly, conventional polarization conversion designs require the upper patch geometry to maintain symmetry along the 45° diagonal axis. Furthermore, the four metallic vias in the backing circuit necessitate four distinct upper metal patches. Therefore, we propose the simplest unit cell configuration as illustrated by element 1 in Figure S4(a). To broaden the polarization conversion bandwidth, we implemented progressive geometric modifications: starting from element 1, we extended the current path length through arrow-shaped patches (element 2), followed by introducing slotted dart-shaped patches with additional capacitance (element 3). This structural evolution gradually increases the number of resonant frequencies from three to five, as shown in Figure S4(b). In addition, the overall co-polarized reflection amplitude $|Rxx|$ of element 3 is smaller than that of elements 1 and 2, except near the 2nd resonance point of element 1, indicating that its overall polarization conversion rate is better. Therefore, the above simulated results indicate that the shape of the proposed DTMM unit helps to increase its polarization conversion bandwidth.

S5. The schematic diagram of DTMM control circuit

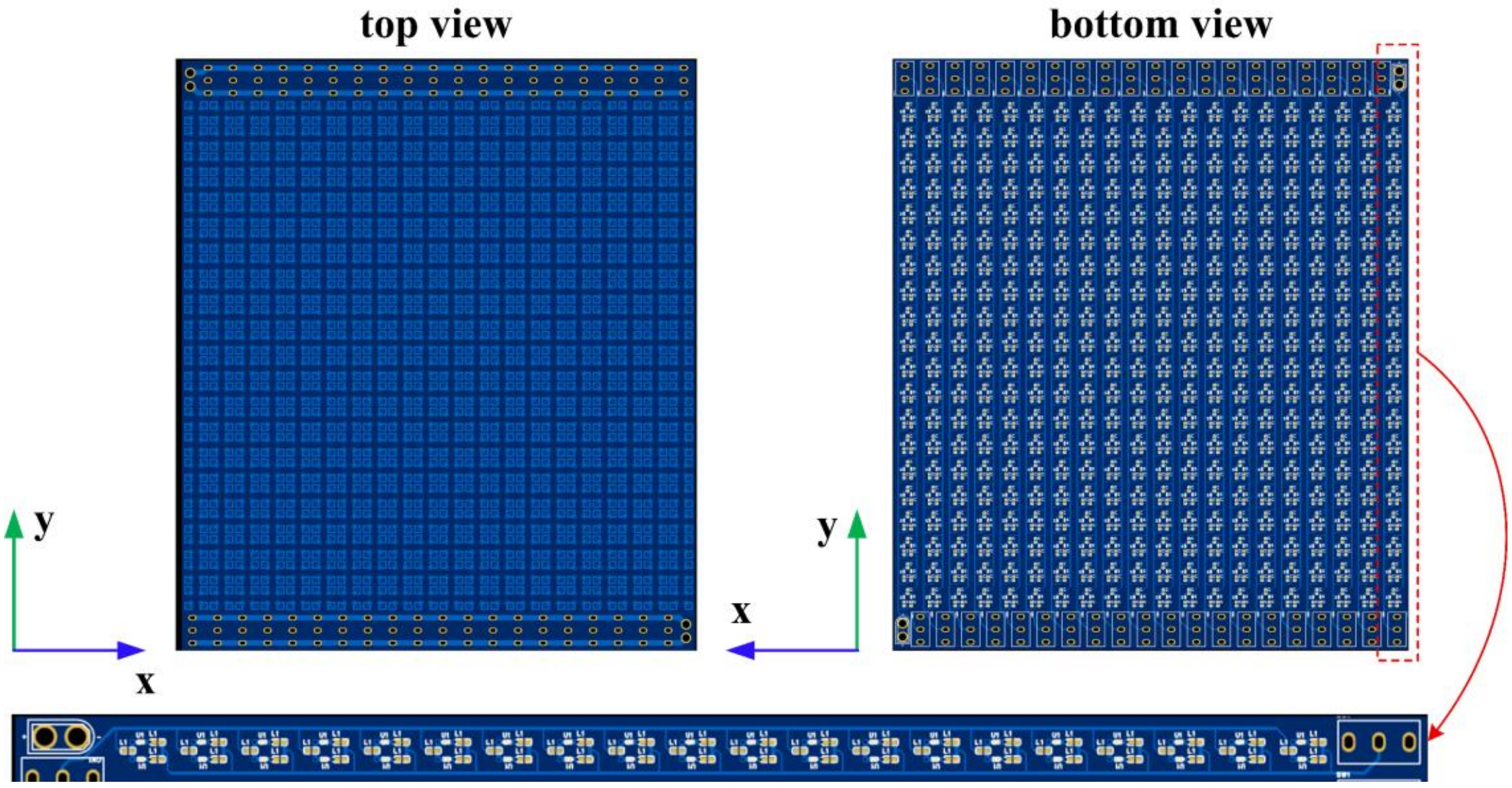

Figure S5. The PCB schematic diagram of the DTMM control circuit.

The PCB fabrication schematic of the proposed metasurface, designed and exported using JLC EDA, is presented in Fig. S5 as an additional illustrative supplement for the control circuit. The entire DTMM is composed of 20×20 unit arrangements. Two PIN diodes within the unit are placed along the y-axis and connected in reverse parallel. The PIN diodes of all units in the same column are connected in parallel on two vertical buses, with the microstrip line on the upper side of the diode connected to the right bus and the microstrip line on the lower side connected to the left bus. On all columns, the right vertical bus is connected in parallel with the bias bus above the unit to connect the input voltage V_1, and the left vertical bus is connected in parallel with the bias bus below the unit to connect the input voltage V_2. Based on this control circuit structure, centralized and unified control of all units can be achieved, and the requirement for high input voltage is reduced. In addition, by controlling the input voltages V_1 and V_2, the DTMM unit can achieve flexible control over multiple functional states, including continuous tuning of the reflection amplitude between two orthogonal polarizations and 1-bit reflection phase switching under polarization conversion.

S6. Performance comparisons between the proposed DTMM and existing designs

Table S1. Comparisons between the proposed DTMM and existing designs

Ref.	Unit capabilities			Bandwidth	Dual-pol	Phase resolution	Design complexity
	Amp.	Pha.	Pol.				
[R3]	×	√	×	5.2-9.4GHz 57.5%	×	180°±20°	high
[R4]	×	√	×	10.5-17.5GHz 50%	×	180°±20°	high
[R5]	√	√	×	9.5-11.5GHz 19%	×	180°±20°	high
[R6]	√	√	×	8-13GHz 47.6%	×	Not given	easy
[R7]	×	√	√	7.2-7.8GHz 8%	√	180°±25°	medium
[R8]	√	√	√	6.5-10.2GHz 44.3%	√	Not given	high
**Ours**	√	√	√	**7-13GHz 60%**	√	**180°±5°**	**medium**

In the comparison Table S1, we have also strengthened the comparison with existing designs. Existing reconfigurable metasurfaces mostly control only two EM properties, with rarely reported cases demonstrating simultaneous amplitude, phase, and polarization control. Additionally, conventional reconfiguration approaches typically rely on active components and intricate biasing networks, whose parasitic effects limit the unit bandwidth, particularly the phase bandwidth. To enhance phase bandwidth, complex interdigital capacitors, magneto-electric dipoles, and antenna-circuit configurations are introduced into the unit design, which is time-consuming and computationally intensive. In contrast, the DTMM proposed in this work achieves simultaneous multifunctional control of polarization, amplitude, and phase using a simple method. Its working bandwidth is 7-13GHz, with the maximum relative bandwidth. And the phase control range is controlled at 180°±5°, with the highest accuracy. The above comparison results fully demonstrate the practical application advantages of the proposed DTMM.

S7. Corresponding relationship between modulation depth and control voltage in co-modulation of RCS and HRRP

Table S2. Corresponding relationship between modulation depth and control voltage

Co-polarized modulation depth Δx_{co}	Bias voltage difference U_d (HH/VV)	Cross-polarized modulation depth Δx_{cross}	Bias voltage difference U_d
0	0/0	0	0
0.1	0.65/0.67	0.1	0.70
0.2	0.82/0.85	0.2	0.84
0.3	0.94/0.95	0.3	0.94
0.4	1.08/1.10	0.4	1.07
0.5	1.16/1.19	0.5	1.17
0.6	1.20/1.22	0.6	1.25
0.7	1.25/1.26	0.7	1.31
0.8	1.28/1.29	0.8	1.34
0.9	——	0.9	——
1	——	1	——

In practical experiments, the control voltage corresponding to the required modulation depth is determined through calibration and mapping lookup tables. Since the forward-biased PIN diode is nonlinear and its equivalent resistance depends on the actual bias current, soldering parasitics, and bias-network loading, the voltage–amplitude relation is calibrated after fabrication rather than obtained only from an analytical diode model. First, the HRRP results of the DTMM on HH, VV, VH/HV channels are measured under a series of voltage differences U_d without time modulation. Then, the measured co-polarized and cross-polarized reflection amplitudes are converted into the modulation depths Δx_{co} and Δx_{cross}. For the co-polarized channel, Δx_{co} is defined by the reduction of the largest co-polarized reflection amplitude relative to the unbiased state U_d=0V. For the cross-polarized channel, Δx_{cross} is defined by the reduction of the largest cross-polarized reflection amplitude relative to the biased state U_d=1.34V. A voltage–modulation-depth lookup table is then constructed as shown in Table S2, and the required voltage for a desired modulation depth is obtained by lookup or interpolation.

S8. Experimental HRRP results based on the DTMM at edge frequencies

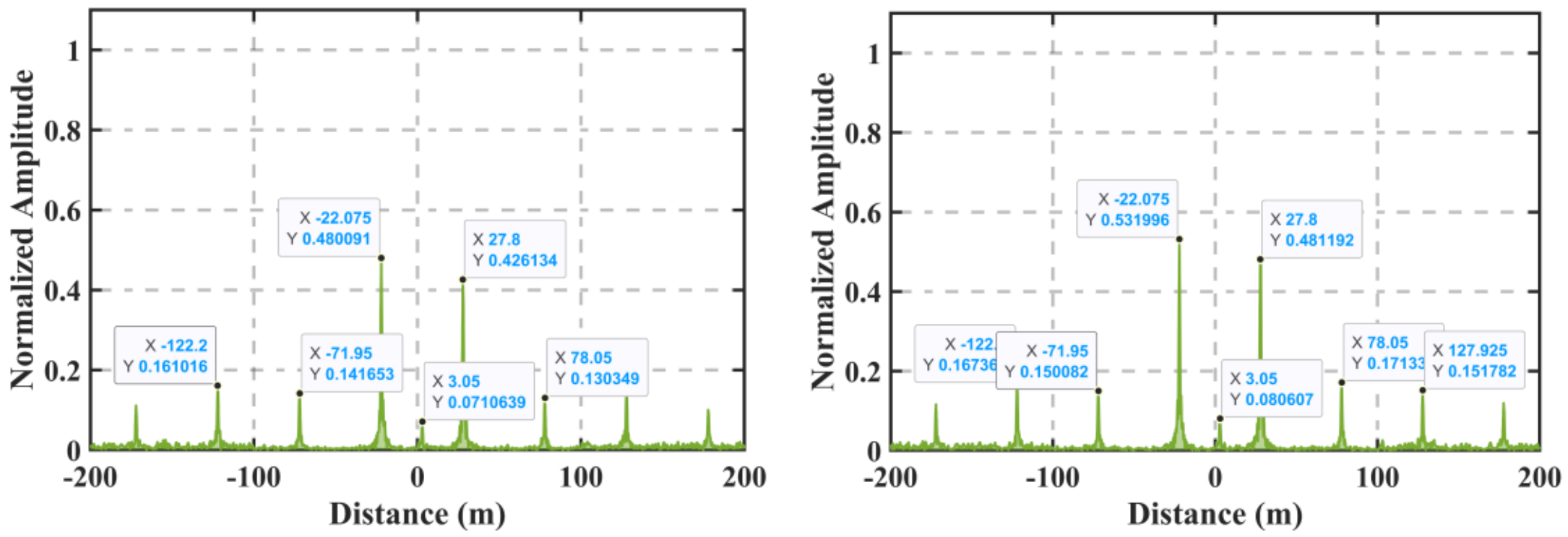

(a) (b)

Figure S6. Experimental cross-polarized HRRP results based on periodic phase time modulation at edge frequencies of (a) 8 GHz and (b) 12 GHz.

To verify the broadband RCS and HRRP co modulation performance of DTMM, we conducted periodic phase time modulation in the cross polarized channel at edge frequencies of 8 GHz and 12 GHz. The actual measurement results are shown in Figure S6. The fixed modulation frequency is 50 kHz, and the cross-polarized modulation depth is 0.8. Even at edge frequencies, the designed DTMM still achieves symmetric false target generation, with adjacent false targets spaced nearly 50m apart, satisfying the calculation formula. The true target amplitude is effectively suppressed. Comparing the 10GHz results shown in Figure 8(g) and (h) of the original manuscript, it is not difficult to find that the true target amplitude is very close to the false target amplitude, effectively verifying the broadband time modulation performance of DTMM.

S9. Comparisons of different RCS and HRRP modulation techniques based on programmable metasurfaces

Table S3. Comparisons of different RCS and HRRP modulation techniques based on programmable metasurfaces

Ref.	Modulated parameters		Coding method	Pol. capability	Co-modulation	Adversarial effect
	Amp.	Pha.				
[R9]	√	×	static	dual	RCS	F1, F2
[R10]	×	√	static	single	RCS	F1, F2
[R11]	√	×	periodic	single	HRRP	F3
[R12]	√	×	nonperiodic	single	HRRP	F4
[R13]	×	√	periodic	single	HRRP	F1, F3
[R14]	×	√	nonperiodic	single	HRRP	F1, F4
[R15]	√	×	periodic	two	HRRP	F3
[R16]	√	√	periodic	single	HRRP	F1, F2, F3
**Ours**	√	√	**periodic+ nonperiodic**	**four**	**RCS+HRRP**	**F1, F2, F3, F4**

F1: target stealth, F2: target camouflage, F3: false target generation, F4: Interference strip obstruction

In Table S3, we present a comprehensive comparison of existing RCS and HRRP manipulation techniques. From the comparison, it is evident that, in terms of functionality, time-domain modulation is typically confined to amplitude or phase, operates in either periodic or nonperiodic patterns, and rarely explores diverse modulation schemes. In terms of polarization, most metasurfaces operate only under single linear polarization (HH or VV). Even the few designs attempting dual-channel control (HH and VV) often fail to address the complete manipulation of complete full polarization scattering matrix (HH, HV, VH, VV) required to deceive fully polarimetric radars. Furthermore, although RCS and HRRP represent the most critical radar signatures for target detection, their coordinated manipulation remains unexplored. Consequently, previous works have only been able to realize one to three radar countermeasure functions. In contrast, our work proposes and demonstrates a co-modulation technique for full-polarimetric RCS and HRRP based on the proposed DTMM. By combining periodic and aperiodic temporal modulation of both amplitude and phase, this technique achieves stealth and camouflage of real targets as well as the generation of discrete false targets and continuous jamming strips, thereby advancing radar countermeasure technology toward multifunctional and multi-dimensional manipulation.

In an ECM scenario, the measured HRRP modulation results can be interpreted as different types of radar-detection interference. Reducing the central target peak decreases the apparent

RCS and may weaken target detectability. Generating periodic false targets introduces additional range-domain peaks, which may mislead range estimation or scattering-center extraction. Producing pseudorandom interference strips masks the local range profile around the real target and may degrade HRRP-based recognition. The additional phase time modulation in the cross-polarized channel further suppresses the real target peak, providing a self-blanking effect. These effects can be reconfigured by changing the temporal coding sequence, modulation depth, and modulation frequency, which highlights the flexibility and adaptability of the DTMM compared with fixed passive jamming structures.

References

[R1]R. H. Caverly, "Microwave and RF p-i-n diode model for time-domain simulation," *IEEE Transactions Microwave Theory and Tech*niques 60, no. 7 (2012): 2195024.
https://doi.org/10.1109/TMTT.2012.2195024

[R2]MACOM Technology Solutions Inc., "Solderable AlGaAs Flip Chip PIN," MADP-000907-14020x, Rev. V8, Datasheet DC-0004063. Accessed: May 25, 2026. [Online], Available.
https://cdn.macom.com/datasheets/MADP-000907-14020x.pdf

[R3]P. Li, H. Yu, J. Su, L. Song, Q. Guo and Z. Li, "A Low-RCS Multifunctional Shared Aperture with Wideband Reconfigurable Reflectarray Antenna and Tunable Scattering Characteristic," *IEEE Transactions on Antennas and Propagation* 71, no. 1 (2023): 3225588.
https://doi.org/10.1109/TAP.2022.3225588

[R4]W. Wu, K. -D. Xu, Q. Chen, T. Tanaka, M. Kozai and H. Minami, "A Wideband Reflectarray Based on Single-Layer Magneto-Electric Dipole Elements with 1-bit Switching Mode," *IEEE Transactions on Antennas and Propagation* 70, no. 12 (2022): 3209693.
https://doi.org/10.1109/TAP.2022.3209693

[R5]Y. Mu, D. Xia, J. Han, et al., "Time-space-coding radiation-stealth metasurface with amplitude-phase co-modulation," *Advanced Functional Materials* 34, no. 46 (2024): 2307408.
https://doi.org/10.1002/adfm.202407802

[R6]H. L. Wang, Y. K. Zhang, T. Y. Zhang, et al., "Broadband and programmable amplitude-phase-joint-coding information metasurface," *ACS Applied Materials & Interfaces* 14, no. 25 (2024): 2c05907.
https://doi.org/10.1021/acsami.2c05907

[R7]B. Liu, J. Du, X. Jiang, et al., "All-in-one integrated multifunctional broadband metasurface for analogue signal processing, polarization conversion, beam manipulation, and near-field sensing," *Advanced Optical Materials* 10, no. 20 (2022): 2201217.
https://doi.org/10.1002/adom.202201217

[R8]Z. Guo, Y. Zhou, H. Yang, et al., "Programmable multifunctional metasurface for polarization, phase, and amplitude manipulation," *Optics Express* 31, no. 21 (2023): 503200.
https://doi.org/10.1364/OE.503200

[R9]H. Li, F. Costa, Y. Wang, Q. Cao, and A. Monorchio, "A wideband multi-functional absorber/reflector with polarization-insensitive performance," *IEEE Transactions on Antennas and Propagation* 68, no. 6 (2020): 2957709.
https://doi.org/10.1109/TAP.2019.2957709.

[R10] H. Xu, D. Guan, B. Peng, Z. Liu, S. Yong, and Y. Liu, "Radar One-Dimensional Range Profile Dynamic Jamming Based on Programmable Metasurface," *IEEE Antennas and Wireless Propagation Letters* 20, no. 10 (2021): 3098698.
https://doi.org/10.1109/LAWP.2021.3098698

[R11] J. Wang, D. Feng, Z. Xu, Q. Wu and W. Hu, "Time-Domain Digital-Coding Active Frequency Selective Surface Absorber/Reflector and Its Imaging Characteristics," *IEEE Transactions on Antennas and Propagation* 69, no. 6 (2021): 3037757.
https://doi.org/10.1109/TAP.2020.3037757

[R12] J. Wang, D. Feng, Y. Kong, S. Quan and S. Xing, "Imaging Properties of Nonperiodic Time-Varying Active Frequency Selective Surface," *IEEE Transactions on Antennas and Propagation* 70, no. 7 (2022): 3161385.
https://doi.org/10.1109/TAP.2022.3161385
[R13] S. Yu, J. Guo, D. Guan, Z. Liu, and Y. Liu, "Information Metasurface-Enabled Dynamically Reconfigurable Radar Electromagnetic Phantoms," *Laser & Photonics Reviews* (2026): 3385421.
https://doi.org/10.1002/lpor.202502619
[R14] J. Guo et al., "Radar Target HRRP Imitation Based on Nonperiodic Time-Coding Digital Metasurface," *IEEE Transactions on Antennas and Propagation* 73, no. 6 (2025): 3537689.
https://doi.org/10.1109/TAP.2025.3537689
[R15] R. Sui, J. Wang, G. Sun, Z. Xu and D. Feng, "Radar Target HRRP Imitation Based on Nonperiodic Time-Coding Digital Metasurface," *IEEE Transactions on Antennas and Propagation* 73, no. 6 (2025): 3526901.
https://doi.org/10.1109/TAP.2025.3526901
[R16] B. Qian, H. Zhao, H. Chu and Y. Guo, "A Dual-Polarimetric High Range Resolution Profile Modulation Method Based on Time-Modulated APCM," *IEEE Transactions on Antennas and Propagation* 73, no. 2 (2025): e02619.
https://doi.org/10.1109/TMTT.2025.3538569
